# Predicting Fear Extinction in Posttraumatic Stress Disorder

**DOI:** 10.3390/brainsci13081131

**Published:** 2023-07-28

**Authors:** Michael W. Lewis, Christian A. Webb, Manuel Kuhn, Eylül Akman, Sydney A. Jobson, Isabelle M. Rosso

**Affiliations:** 1 Center for Depression, Anxiety, and Stress Research, McLean Hospital, Belmont, MA 02478, USA; 2 Department of Psychiatry, Harvard Medical School, Boston, MA 02115, USA

**Keywords:** posttraumatic stress disorder, machine learning, fear extinction, psychophysiology, skin conductance, startle, electrocardiography, penalized regression

## Abstract

Fear extinction is the basis of exposure therapies for posttraumatic stress disorder (PTSD), but half of patients do not improve. Predicting fear extinction in individuals with PTSD may inform personalized exposure therapy development. The participants were 125 trauma-exposed adults (96 female) with a range of PTSD symptoms. Electromyography, electrocardiogram, and skin conductance were recorded at baseline, during dark-enhanced startle, and during fear conditioning and extinction. Using a cross-validated, hold-out sample prediction approach, three penalized regressions and conventional ordinary least squares were trained to predict fear-potentiated startle during extinction using 50 predictor variables (5 clinical, 24 self-reported, and 21 physiological). The predictors, selected by penalized regression algorithms, were included in multivariable regression analyses, while univariate regressions assessed individual predictors. All the penalized regressions outperformed OLS in prediction accuracy and generalizability, as indexed by the lower mean squared error in the training and holdout subsamples. During early extinction, the consistent predictors across all the modeling approaches included dark-enhanced startle, the depersonalization and derealization subscale of the dissociative experiences scale, and the PTSD hyperarousal symptom score. These findings offer novel insights into the modeling approaches and patient characteristics that may reliably predict fear extinction in PTSD. Penalized regression shows promise for identifying symptom-related variables to enhance the predictive modeling accuracy in clinical research.

## 1. Introduction

Laboratory fear extinction studies have provided the mechanistic basis of “gold-standard” exposure therapies for posttraumatic stress disorder (PTSD; [1]), but as many as half of patients do not recover [2]. Psychophysiological measures are promising clinical tools for predicting fear extinction and eventually informing extinction-based exposure treatments [3]. However, to fulfill this promise, novel statistical approaches may be needed to increase the accuracy and generalizability of fear extinction predictions [3,4,5]. Numerous clinical and self-report measures have been associated with fear extinction in some studies, but their findings have not consistently replicated [4,6,7,8]. Thus, it may be beneficial to apply novel bottom-up statistical approaches to simultaneously evaluate multiple predictors of fear extinction in a single study [4,9,10]. Penalized regressions are a class of machine learning approaches used in clinical psychology research to increase predictive accuracy, improve generalizability, and select predictors [11,12,13]. Additionally, prior studies have shown that penalized regression analyses can be combined with complementary OLS regression analyses to identify promising predictors in clinical research [13,14]. However, we are not aware of any studies that have applied penalized regressions, with or without complementary OLS regression analyses, to predict fear extinction in PTSD samples.

Despite the clear clinical relevance of fear extinction in PTSD treatment [15], there is no consensus regarding whether current PTSD diagnoses or symptom severity robustly predict fear extinction [7,9]. For example, some studies have found evidence of deficient fear extinction learning in PTSD patients versus controls, while others have not (as reviewed by [7]). The findings have been similarly mixed when examining PTSD symptom severity [16,17] and specific PTSD symptom clusters [16,18,19,20] as predictors of fear extinction. Several explanations for these inconsistent findings have been proposed, including: the biological [18,21] and clinical [21,22] heterogeneity of PTSD, the insufficient statistical power of previous studies [7], and the methodological heterogeneity across previous studies [9,23]. These findings align with prior calls for an increased emphasis on the within-group differences in fear extinction in PTSD [21,22]. Further, they highlight a need for strategies to improve predictive performance and generalizability [7,9,23]. Ultimately, the accurate and generalizable prediction of fear extinction in PTSD samples may inform advances in precision extinction-based treatments [3,15].

Prior evidence has suggested that individual differences in demographic, self-reported, clinical, and psychophysiological characteristics may predict fear extinction, but specific robust predictors have yet to be identified (for reviews, see [4,6,24]). Each of the following variables has been associated with fear extinction, albeit inconsistently across studies: sleep (for reviews, see [25,26,27]), sex [6,8,17,28], level and type of childhood trauma exposure [29,30], level of adulthood trauma exposure [19,31], severity of depression symptoms [32,33], trait anxiety (reviewed by [6]), state anxiety (reviewed by [6]), and resting heart rate variability (HRV) [24,34,35]. Although the literature thus far is inconclusive, each of these patient characteristics is relevant to PTSD treatment and they warrant further study as predictors of fear extinction.

Given the lack of consensus on predictors of fear extinction, it is important to consider individual differences associated with fear physiology and PTSD that have not (to our knowledge) specifically been examined in relation to extinction. A number of variables have been associated with fear physiology and PTSD, including trait dissociation [36,37], a dissociative clinical subtype of PTSD [37], trait resilience [38,39], anhedonia symptoms [40,41,42], trauma exposure type [43,44], anxiety sensitivity [45,46,47,48,49,50,51], trait fearfulness [52], age [6], race [53], baseline heart rate [54,55], and baseline startle [3,56,57]. Although we are not aware of any studies examining these variables in relation to fear extinction specifically, this evidence suggests that these patient characteristics may be relevant to fear extinction in PTSD. For additional details, see the Supplementary Materials.

Physiological activity during stressors that occur before fear extinction learning also may predict extinction in PTSD. One such stressor is the dark-enhanced startle paradigm, which elicits physiological responses in an anxiety-provoking environment (i.e., an unfamiliar dark room) [58]. Alterations in parasympathetic (e.g., HRV) and sympathetic (e.g., heart rate) activity evoked by dark-enhanced startle tasks have been associated with PTSD and fear extinction [58]. Similarly, fear acquisition, a pre-requisite for de novo laboratory fear extinction [1], has been associated with physiological responses during extinction in some PTSD studies [19,59]. Notably, in PTSD samples, responding to a safety cue (CS−) during fear acquisition may be a better predictor of extinction than physiological responses to a danger cue (CS+), suggesting a relationship between deficient safety inhibition and impaired fear extinction in PTSD [59,60]. In addition to startle, skin conductance response (SCR) evoked by a CS+ and CS− and individual differences in continuously measured (i.e., tonic) heart rate and HRV during acquisition are candidate predictors of extinction in PTSD [59,60,61,62]. Thus, all these physiological variables hold promise as predictors of extinction in PTSD.

Approaches that can consider a range of potential predictors for fear extinction include novel multivariable statistical techniques such as penalized regressions. Applying a data-driven approach that explores numerous variables in a single study may be more impactful than a series of studies that examine one to a few at a time [4]. Penalized regressions are machine learning algorithms that enable the simultaneous modeling of many predictors [63,64]. As such, these models hold promise for improving the accuracy and generalizability of multivariable predictions of fear extinction. Ridge Regression, Lasso Regression, and Elastic Net Regression (ENR) are three commonly used penalized regression models that have advanced the clinical research in other domains [65,66,67]. In a study of depression treatment outcomes, each was found to be more accurate than traditional Ordinary Least Squares (OLS) regression in both a training sample and separate holdout sample [11]. This suggests that penalized regression algorithms can increase the accuracy and generalizability of clinical predictions [11]. One advantage of penalized regression versus OLS regression is the use of regularization to address multicollinearity: this enables the development of multivariable models that can account for many intercorrelated predictor variables [63]. A second advantage is that Lasso and ENR perform variable selection by including a penalty term that drops some variables from the model [11,13,68]. The penalty term allows Lasso and ENR to select the subset of candidate predictors that minimize the prediction error [11,13,68]. Thus, in addition to an increased predictive accuracy and generalizability, Lasso and ENR are useful tools for selecting a parsimonious set of predictor variables. For example, a prior study on depression treatment applied ENR to select the predictors of post-treatment outcomes [11]. This study investigated 51 pre-treatment patient characteristics and identified a subset of 14 as predictors using ENR [11], demonstrating that ENR can identify a parsimonious subset of predictor variables from a wide array of potential predictors [11]. Similarly, ENR has previously been used to select an optimal multivariable model to prospectively predict PTSD symptoms using imaging, demographic, and clinical patient characteristics [14]. Moreover, simulation studies have demonstrated that Lasso and ENR perform more accurate and parsimonious variable selection than other procedures, such as step-wise variable selection [69,70,71]. Thus, previous work has provided proof-of-concept for combining penalized regressions with complementary univariate and multivariable OLS regression analyses to identify predictors. To our knowledge, no study has yet applied this strategy to predict fear extinction in PTSD.

The aims of this study were to: (1) compare the accuracy and generalizability of penalized regression (Ridge, Lasso, and ENR) and conventional OLS regression for predicting fear extinction in a sample of trauma-exposed adults with a range of PTSD symptoms, and (2) identify the clinical, psychological, and physiological characteristics that predict fear extinction. Informed by the theory and evidence suggesting that FPS during specific extinction phases may be a promising translational measure of fear extinction in PTSD samples [19,72], we used FPS during early extinction and late extinction as our outcome variables. Based on evidence that penalized regression approaches may improve the predictive accuracy in a cross-validated training sample and the generalizability to a holdout sample [11,65,67], we hypothesized (Primary Hypothesis 1) that our three penalized regression approaches would be more accurate than conventional OLS regression in both the cross-validated training sample and a separate holdout sample. We also compared our three penalized regression approaches, but did not have a specific hypothesis regarding the most accurate. Based on evidence that startle variables are highly intercorrelated [73], we hypothesized (Primary Hypothesis 2) that startle variables would be significant predictors of FPS during both early and late extinction. We regarded the comparisons between specific startle variables (e.g., baseline startle versus dark-enhanced startle) as exploratory. Given the lack of consensus regarding predictors of fear extinction [6,10], we regarded all non-startle predictor variables as exploratory.

## 2. Materials and Methods

### 2.1. Participants

The participants were 125 trauma-exposed adults recruited from the greater Boston metropolitan area. The inclusion criteria were: the ability to provide written informed consent, being aged between 18–55, exposure to at least one DSM-5 PTSD criterion A trauma, and meeting the criteria for at least 2 PTSD symptom clusters, as defined by the Clinician-Administered PTSD Scale for DSM-5 (CAPS; [74]). The exclusion criteria were: a medical condition that would confound the results, history of head trauma, current treatment with an antipsychotic, benzodiazepine use within 48 h, moderate-to-severe alcohol or substance use disorder in the past month, a current psychotic disorder, current anorexia, current obsessive-compulsive disorder, a current manic or mixed mood episode, and a lifetime history of schizophrenia or schizoaffective disorder. The Mass General Brigham and Partners Human Research Committee approved the study procedures. All the participants provided written informed consent. Table 1 shows the participant demographic and clinical characteristics.

### 2.2. Procedures

After providing informed consent, the participants filled out self-report questionnaires and completed clinical interviews administered by doctoral-level psychologists. During a laboratory visit, the participants then completed a dark-enhanced startle task consisting of three phases, as previously described [58]. After the dark-enhanced startle, the participants completed a fear conditioning paradigm, as previously described [16].

#### 2.2.1. Clinical Interviews

All the participants were administered the CAPS for DSM-5 [74] and the Mini International Neuropsychiatric Interview (MINI; [75]) by doctoral-level psychologists.

The CAPS [74] is the gold-standard semi-structured interview for PTSD assessment [76] and was used to determine a diagnosis of PTSD (Table 1). In addition, we derived the CAPS total score and scores for each of the four symptom clusters (B-E), due to prior evidence that the relationship between PTSD symptom severity and extinction may depend on the PTSD symptom cluster examined [16,18,19,20]. The MINI was used to assess other DSM-5 disorders, including those relevant to the exclusion criteria above.

#### 2.2.2. Self-Report Measures

Table 2 shows the full list of the self-report measures that we examined. Some of these can be used to derive both total and subscale scores. Based on evidence and theory from the literature, we included a combination of total and/or subscale scores for several measures (see the list below and a detailed justification behind each decision in the Supplementary Materials).

##### Trauma-Exposure and PTSD Symptom Questionnaires

The Childhood Trauma Questionnaire (CTQ; [77]) is a 28-item questionnaire used to assess the occurrence and frequency of childhood abuse and neglect. We included the CTQ total score and each of the 5 CTQ subscale scores: Emotional Abuse, Emotional Neglect, Physical Abuse, Physical Neglect, and Sexual Abuse. The Life Events Checklist (LEC; [78] is a self-report measure of exposure to 16 types of potentially traumatic events. We included the LEC Experienced + Witnessed and LEC Experienced scores. The PTSD Checklist for DSM-5 (PCL-5; [79]) is a 20-item self-report measure that assesses the 20 DSM-5 symptoms of PTSD. We included the PCL-5 total score in our main analyses and the PCL-5 cluster scores in our post hoc analyses (see Section 3.3).

##### Dissociation Questionnaires

The Dissociative Experiences Scale-II [80] is a 28-item measure that assesses both normative (e.g., daydreaming) and clinically significant dissociative experiences during daily life. We included the Dissociative Experiences Scale total score and its 3 subscale scores: amnesia, absorption, and depersonalization/derealization. The Multiscale Dissociation Inventory (MDI; [81]) is a 30-item self-report measure of clinically impairing dissociative symptoms. Although we did not have sufficient MDI data to include the MDI in our primary analyses (see Section 3.3 and Supplementary Materials for details), our post hoc analyses used the MDI total score and 6 MDI subscale scores: disengagement, depersonalization, derealization, emotional constriction/numbing, memory disturbance, and identity dissociation (see Section 3.3 for details).

##### Depression Questionnaires

The Beck Depression Inventory-II (BDI; [82]) is a 21-item inventory of depressive symptom severity. We included the BDI total score. The Snaith–Hamilton Pleasure Scale (SHAPS; [83]) is a 14-item anhedonia symptom questionnaire. We included the SHAPS total score.

##### Fear and Anxiety Questionnaires

The State-Trait Anxiety Inventory (STAI; [84]) is a 40-item scale designed to measure trait and state anxiety, and we included both subscale scores. The Fear Survey Schedule-II (FSS; [85]) is a 51-item questionnaire assessing the tendency to experience fear in response to various real-world stressors and stimuli. We included the FSS total score. The Anxiety Sensitivity Index-3 (ASI; [86]) is an 18-item questionnaire assessing fear of anxiety symptoms. We included the ASI total score.

##### Sleep and Resilience Questionnaires

The Pittsburgh Sleep Quality Index (PSQI; [87]) is a 9-item questionnaire on sleep quality and patterns. We included the PSQI total score. The Connor–Davidson Resilience Scale (CDRISC; [88]) is a 10-item questionnaire assessing the dispositional tendency to respond to stress and adversity with resilience. We included the CDRISC total score.

#### 2.2.3. Laboratory Paradigms

##### Dark-Enhanced Startle

We used a dark-enhanced startle paradigm, as previously published [58]. First, during a 2 min *Baseline* period, the participants acclimated to the laboratory environment and no startle probes occurred. Second, during a 2 min *Habituation* phase, 8 startle probes were delivered with the lights on. Third, during a 4 min *Dark-Light* phase, the participants experienced 4 1 min blocks of alternating dark and light, with the order of dark and light counterbalanced between the subjects. Each dark block and light block included 4 startle probes. Across the entire dark-enhanced startle task, the inter-trial intervals ranged from 10 to 30 s.

##### Fear Conditioning

We used a fear conditioning and extinction paradigm, as previously published [16]. Briefly, this paradigm consisted of three phases. First, *Habituation* included 7 Noise Alone (NA) trials, 4 CS+ trials, and 4 CS− trials. Second, *Acquisition* included 12 NA trials, 12 CS− trials, and 12 CS+ trials, with a US presented 0.5 s after the CS+ termination with 100% reinforcement. Third, *Extinction* included 16 NA trials, 16 CS− trials, and 16 CS+ trials, with no US presentation (0% reinforcement). *Acquisition* and *Extinction* were pseudorandomized and counterbalanced between the subjects; the trials were presented in blocks, with each block containing 4 trials of each type. All the trial durations were 6 s, and each trial included an auditory startle probe. The auditory startle probes were 106 dB 40 ms white noise bursts with a near-instantaneous rise/fall time delivered 5.6 s into each trial. The inter-trial intervals varied between 9 and 22 s. The stimuli were: a 140 psi airblast delivered to the larynx, which served as the unconditioned stimulus (US), and colored shapes displayed against a white background, which served as the conditioned stimuli. The NA trials consisted of only the white computer screen and an auditory startle probe.

#### 2.2.4. Physiological Data Acquisition and Processing

##### Startle

Electromyography (EMG) was continuously recorded from two 5 mm Ag/AgCl electrodes filled with electrolyte gel and attached below the right eye. The EMG data were acquired at a sampling rate of 1000 kHz, amplified, and digitized using the EMG module of the Biopac MP150 (Biopac Systems, Inc., Aero Camino, CA, USA). The EMG signal was filtered with low- and high-frequency cut-offs at 28 and 500 Hz, respectively. The reflexive eyeblinks to all the startle probes within a response window from 20 to 120 milliseconds were quantified by calculating the difference in the amplitude between the peak of the response and the EMG value at the response onset [89]. Based on prior recommendations [89], the individual startle probes were examined, and invalid trials (i.e., blinks in which there was excess noise, blinks which began prior to the latency window, or trials in which a spontaneous blink occurred immediately before the startle probe) were removed. Specifically, 5.47% of individual startles were deleted and treated as missing.

For each trial during *Acquisition* and *Extinction*, the FPS was calculated by subtracting the startle magnitude to the corresponding noise-alone trial from the startle magnitude to the conditioned stimulus (e.g., CS+ or CS−) trial. Thus, the FPS reflected the degree to which the reflexive startle response elicited by the startle probe was elevated when in the presence of the CS+ or CS−, relative to when no conditioned stimulus was present. For each block during *Acquisition* and *Extinction*, the mean FPS was calculated across all the trials in the block. The dark-enhanced startle response was calculated by subtracting the mean startle magnitude during the light condition of the dark-enhanced startle from the mean startle magnitude during the dark condition of the dark-enhanced startle. The baseline startle response was calculated by taking the mean startle magnitude across all 7 *Habituation* trials that occurred before the dark-enhanced startle.

##### Skin Conductance

Skin conductance was continuously recorded from two 5 mm Ag/Cl electrodes filled with isotonic paste and attached to the palm of the non-dominant hand. The skin conductance data were acquired at a sampling rate of 1000 Hz, amplified, and digitized using the Galvanic Skin Response module of the Biopac MP150 (Biopac Systems, Inc., Aero Camino, CA, USA). The SCR to all the conditioned stimuli within a response window from 0.9 to 6 s after the stimulus onset was quantified by calculating the difference in the skin conductance level between the response peak and the response trough [73,90]. The SCR to each trial was square root transformed. Based on recommendations, [73,90], each individual SCR trial was examined, and invalid SCRs (i.e., excessive noise) were treated as missing. SCR trials for which no detectable response occurred (i.e., the recorded amplitude was less than the minimum detectable amplitude of 0.2 μS) were treated as non-responses with an amplitude of 0. Applying these criteria, 6.93% of individual SCRs were deleted and treated as missing.

For each block during *Acquisition*, the SCR difference score was calculated by subtracting the mean SCR response to the CS− trials from the mean SCR response to the CS+ trials. Additionally, the SCR habituation was calculated by taking the mean SCR across all the *Habituation* trials before *Acquisition*.

##### Cardiography

Electrocardiography (ECG) was continuously recorded from three 11 mm Ag/AgCl electrodes filled with electrolyte gel and attached under each clavicle and on the left forearm. The ECG data were acquired at a sampling rate of 1000 Hz, amplified, and digitized using the Galvanic Skin Response module of the Biopac MP150 (Biopac Systems, Inc., Aero Camino, CA, USA). For all the physiological tasks, the heart rate and HRV features were extracted from 1 min intervals of continuous ECG data. Automated QRS detection was performed in MindWare [91], and errors were detected using visual screening and corrected manually. Based on recommendations [91], each 1 min segment of the ECG data was examined for erroneous R peaks and cardiac arrhythmia. Any segment with greater than 10% erroneous R peaks or greater than 2% cardiac arrhythmia was treated as missing. Applying these criteria, 15.20% of individual ECG segments were deleted and treated as missing.

The baseline heart rate and HRV were calculated as the mean of the heart rate and HRV across all the 1 min segments during *Habituation* prior to the dark-enhanced startle. The dark-enhanced heart rate and dark-enhanced HRV were calculated by subtracting the mean of the heart rate across all the 1 min segments during light and the HRV across all the 1 min segments during light, respectively, from the mean of the heart rate across all the 1 min segments during dark and the HRV across all the 1 min segments during dark, respectively. The heart rate during *Acquisition* and HRV during *Acquisition* were calculated by taking the average across all the 1 min segments of the heart rate during *Acquisition* and the HRV during *Acquisition*.

#### 2.2.5. Statistical Analyses

##### Outcome Variables

Given the evidence from previous studies on the importance of considering temporal dynamics when using FPS to measure the fear extinction in PTSD samples [18,19], we followed that precedent to operationalize fear extinction. Specifically, we examined the FPS during both early and late extinction as response variables. Early extinction was calculated as the mean FPS to the CS+ across the first two extinction blocks and late extinction as the mean FPS to the CS+ across the last two blocks of extinction [19].

##### Predictor Variables

The predictor variables included clinical symptom measures, self-report measures, demographic characteristics, baseline psychophysiological measures, psychophysiological measures taken during the dark-enhanced startle task, and psychophysiological measures taken during fear *Acquisition*. See Table 2 for a complete list.

##### Analysis Pipeline

**Figure 1:**
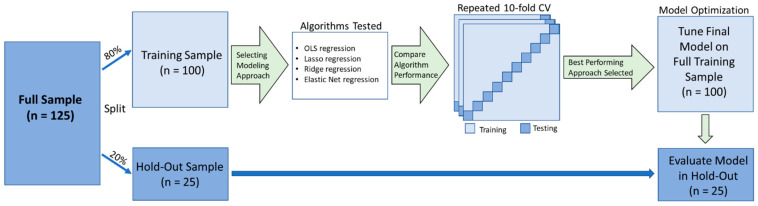
Schematic of the analysis pipeline (adapted from [11]). The total sample (n = 125) was split into a training sample (80%) and a holdout validation sample (20%). We compared the performance of 3 machine learning algorithms (in addition to conventional linear regression) via 100 iterations of 10-fold cross-validation. The best-performing model (lowest mean squared error; MSE) was tuned and implemented (without modification) in the holdout validation sample.

Figure 1 displays a schematic of our analysis pipeline. We split our sample of 125 participants into a training sample and holdout sample. Following precedent [11], the 20% of the participants who most recently visited the lab were assigned to the holdout sample (i.e., a *temporal validation*). The predictor variables were z-transformed. The data preparation and model development only used data drawn from the training sample.

The missing data were imputed separately for the training and holdout samples using the missForest [92] package in R [93]. For the 50 predictor variables in our models, the missingness rates were as follows: 12 variables with 0 missing, 25 variables with less than 5% missing, 5 variables with between 5 and 10% missing, and 10 variables with between 10 and 18% missing. For the outcome variables, all the participants had complete data.

##### Comparing Predictive Models

For both of our response variables (i.e., early extinction and late extinction), we compared the performances of 4 different types of predictive models, each of which was implemented using the glmnet package in R [94] with the wrapper function provided by the caret package in R [95]. We implemented three different types of penalized regression model: least absolute shrinkage and selection operator (Lasso) regression, Ridge regression, and ENR. For comparison, we also applied conventional ordinary least squares (OLS) linear regression. To minimize the overfitting, we used repeated cross-validation with 10 folds and 100 repeats. Importantly, the cross-validation procedure ensured that all the predictions of the FPS during early extinction and the FPS during late extinction for all the participants were generated from models trained without using their own data. Within the cross-validation step, we used the caret package’s resampling grid search to select the optimal tuning hyperparameters, alpha and lambda [95]. Specifically, for ENR, each combination of alpha and lambda was tested (from 0 to 1 by 0.05 increments) and the optimal values were selected (i.e., the values that minimized the MSE in the cross-validated training sample) [95]. The same procedure was applied for the Lasso and Ridge regression, with the exception that Lasso only identified the optimal value for lambda (with alpha fixed at 0) and Ridge only identified the optimal value for alpha (with lambda fixed at 0) [95]. We compared the models’ performances in the testing sample based on the cross-validated mean squared error (MSE). As a lower MSE indicates a lower predictive error, models with a lower MSE are more accurate predictive models. We also examined the mean absolute error (MAE), which measures the predictive error such that a lower MAE indicates less error and a higher predictive accuracy. Finally, we examined the R^2^ (coefficient of determination) values based on the following formula: 1 − [MSE/var(y)]. The R^2^ value provides a measure of predictive accuracy on a standardized scale with a maximum score of 1, but it is not lower bounded [96]. An R^2^ of 1 indicates a perfect predictive performance, an R^2^ of 0 is equivalent to chance, and an R^2^ < 0 indicates a worse predictive performance than chance.

After the cross-validation, all the models were tuned on the entire training sample to derive the final model parameters, which were then used to predict the outcome for the holdout sample. The model performance in the holdout sample was evaluated as described in the previous paragraph.

##### Examining Specific Predictor Variables

For both early extinction and late extinction, we used 8 different criteria to identify the predictor variables: (1) we identified the variables that were significant univariate predictors of extinction at a Bonferroni-corrected threshold of *p* < 0.001, (2) we identified the variables that were significant univariate predictors at a nominal significance threshold of *p* < 0.05, (3) we identified the variables that were retained as predictors in Lasso, (4) we identified the variables that were retained as predictors in ENR, (5) we identified the variables that were statistically significant predictors when including the predictors retained by Lasso in a multivariable regression model and applying a Bonferroni correction based on the number of variables included in the model, (6) we identified the variables that were statistically significant predictors when including the predictors retained by Lasso in a multivariable regression model using a nominal significance threshold of *p* < 0.05, (7) we identified the variables that were statistically significant predictors when including the predictors retained by ENR in a multivariable regression model and applying a Bonferroni correction based on the number of variables included in the model, and (8) we identified the variables that were statistically significant multivariable predictors when including the predictors retained by ENR in a multivariable regression model using a nominal significance threshold of *p* < 0.05.

To evaluate criteria 1 and 2, we performed 100 univariate regressions in the whole study sample. Specifically, we performed 50 univariate regressions to predict early extinction and 50 univariate regressions to predict late extinction (i.e., one univariate regression per predictor variable). The predictor variables were z-transformed before their inclusion in the models. The participants missing the predictor variable for a given univariate model were dropped from the corresponding univariate regression. Criteria 3 and 4 were evaluated based on cross-validated Lasso and ENR performed in the training sample only. Criteria 5–8 were evaluated based on multivariable OLS regressions performed in the whole sample. For these multivariable OLS regressions performed in the whole sample, we used the R default setting, which dropped the participants’ missing data for any predictor from the model. The predictor and response variables were z-transformed before their inclusion in the models, such that the estimated regression coefficients were fully standardized and comparable across the predictors and responses. In addition to our 8 criteria, we also examined the coefficients applied to each variable within each model. Our decision to evaluate the predictor significance at both an uncorrected threshold of *p* < 0.05 and a Bonferroni-corrected threshold was based on the importance of considering both type I and type II errors for exploratory analyses [97]. Currently, there is no clear consensus with regard to *p*-value correction in exploratory research [97]. While some have argued that exploratory analyses should always apply a Bonferroni correction to control for type I errors (e.g., [98,99,100]), others have argued that corrected *p*-values lead to excessive type II errors and have recommended that no correction be applied (e.g., [101,102,103,104]). Thus, to balance these considerations and maximize transparency, we reported the significance of the findings at both corrected and uncorrected thresholds.

##### Post Hoc Analyses

Although our study included the Multiscale Dissociation Inventory (MDI; [81]), this measure was not included in the primary analyses due to the excessive missing data in the training sample (see the Supplementary Materials for details). We initially found that the Dissociative Experiences Scale, and its depersonalization/derealization subscale, predicted early extinction. Following up on this finding, we performed post hoc analyses of the MDI and its subscales.

Finally, we performed a post hoc analysis of self-reported PTSD symptom severity using the PCL-5 [105]. We initially found that PTSD Cluster E scores on the CAPS predicted early extinction; this is consistent with two prior studies that used CAPS [18,20], but inconsistent with two prior studies that used a self-report measure called the PTSD Symptom Scale (PSS) [16,19]. Although we did not have PSS data in this study, we checked to see if a different self-report measure, the PCL-5, would yield a finding consistent with our CAPS finding.

## 3. Results

In the whole sample, the FPS levels were variable during early extinction (range = −37.56–236.9; mean = 52.54; and standard deviation = 48.57) and late extinction (range = −27.07–147.73; mean = 33.46; and standard deviation = 37.04). In the training sample, the FPS had a mean of 53.23 during early extinction (standard deviation = 48.96) and a mean of 32.51 during late extinction (standard deviation = 35.49). In the holdout sample, the FPS had a mean of 49.96 during early extinction (standard deviation = 48.01) and a mean of 37.12 during late extinction (standard deviation = 43.22). See Supplementary Figures S1 and S2 for the distributions of the FPS during early extinction and late extinction.

### 3.1. Model Fit Comparisons

#### 3.1.1. Early Extinction

To predict the FPS during early extinction, the 10-fold repeated cross-validation procedure indicated that the optimal tuning parameters were as follows: Lasso Regression (alpha 1, lambda 3.59); Ridge Regression (alpha 0, lambda 40.37); and ENR (alpha 0.1, lambda 23.55). In the training sample, the cross-validation results indicated that the model with the lowest prediction error was ENR (MSE = 1296.41; MAE = 28.30; and R^2^ = 0.50). Likewise, the model with the lowest prediction error in the holdout sample was ENR (MSE = 726.91; MAE = 21.29; and R^2^ = 0.57). The Conventional OLS Linear Regression was the model with the highest prediction error in both the cross-validated training sample and holdout sample, and was less accurate than chance in the holdout sample (R^2^ = −0.29). For a comparison of the fit indices across all the models for predicting early extinction in the cross-validated training and holdout samples, see Table 3.

#### 3.1.2. Late Extinction

To predict the FPS during late extinction, the 10-fold repeated cross-validation procedure indicated that the optimal tuning parameters were as follows: Lasso Regression (alpha 1, lambda 2.98); Ridge Regression (alpha 0, lambda 48.63); and ENR (alpha 0.1, lambda 17.50). In the training sample, the cross-validation results indicated that the model with the lowest prediction error was ENR (MSE = 966.55; MAE = 23.60; and R^2^ = 0.29). However, the model with the lowest prediction error in the holdout sample was Lasso Regression (MSE = 2037.19; MAE = 32.18; and R^2^ = 0.29). The Conventional OLS Linear Regression was the model with the highest prediction error in both the cross-validated training sample and holdout sample, and was less accurate than chance in the holdout sample (R^2^ = −0.61). For a comparison of the fit indices across all the models for predicting late extinction in both the cross-validated training sample and holdout sample, see Table 4.

### 3.2. Predictors

#### 3.2.1. Early Extinction

Table A1 shows the variables that met at least one criterion used to identify the predictors of early extinction. Dark-enhanced startle was the only variable that met all eight criteria. No variable met seven or six out of the eight criteria. Four variables met five out of the eight criteria: the Depersonalization and Derealization subscale of the Dissociative Experiences Scale, the Severity of CAPS Cluster E Symptoms (i.e., Alterations in Arousal and Reactivity), the FPS to the CS+ during block 1 of the Acquisition, and the FPS to the CS+ during block 3 of the Acquisition. Four variables met four out of the eight criteria: Baseline Startle, the FPS to the CS+ during block 2 of the Acquisition, the FPS to the CS− during block 2 of the Acquisition, and the FPS to the CS− during block 3 of the Acquisition. Three variables met three out of the eight criteria: the total score on the Pittsburgh Sleep Quality Index, the Physical Neglect subscale on the Childhood Trauma Questionnaire, and female sex. Five variables met two out of the eight criteria, ten variables met one out of the eight criteria, and twenty-three variables did not meet any of the eight criteria.

Across the 50 univariate regression analyses performed on the whole sample to predict the FPS during early extinction, 7 were significant at the Bonferroni-corrected significance threshold of *p* < 0.001, and an additional 10 were significant only at the uncorrected (nominal) significance threshold of *p* < 0.05. Across the 50 potential predictor variables included in the cross-validated training sample, Lasso selected 14 predictor variables and ENR selected 23 predictor variables. When including the 14 predictor variables selected by Lasso in a multivariable regression model using the whole sample, 1 variable was significant at the Bonferroni-corrected significance threshold of *p* < 0.00357 (0.05/14), and an additional 3 variables were significant only at the uncorrected significance threshold of *p* < 0.05. When including the 23 predictor variables selected by ENR in a multivariable regression model using the whole sample, 1 variable was significant at the Bonferroni-corrected significance threshold of *p* < 0.00217 (0.05/23), and an additional 6 variables were significant only at the uncorrected significance threshold of *p* < 0.05. For detailed statistics from the univariate and multivariable regression models, see Supplementary Tables S1 and S2. For a heatmap comparing the coefficient weights of all 50 predictors using a simple univariate regression and the four cross-validated machine learning models, see Supplementary Figure S3.

#### 3.2.2. Late Extinction

Table A2 shows the variables that met each criterion used to identify the predictors of late extinction. Across the eight criteria, zero variables met all eight criteria, and zero variables met seven criteria. Baseline startle was the only variable to meet six criteria. Two variables met five criteria: the FPS to the CS+ during block 2 of the Acquisition and the FPS to the CS− during block 3 of the Acquisition. The CAPS Cluster C score was the only variable to meet four criteria. Four variables met three criteria: the Physical Neglect Subscale of the Childhood Trauma Questionnaire, dark-enhanced startle, the FPS to the CS− during block 1 of the Acquisition, and the FPS to the CS− during block 2 of the Acquisition. Ten variables met two criteria, four variables met one criterion, and twenty-eight variables met zero criteria.

Across the 50 univariate regression analyses performed on the whole sample to predict the FPS during late extinction, 4 were significant at the Bonferroni-corrected significance threshold of *p* < 0.001, and an additional 5 were significant only at the uncorrected significance threshold of *p* < 0.05. Across the 50 potential predictor variables included in the cross-validated training sample, Lasso selected 14 predictor variables and ENR selected 22 predictor variables. When including the 14 predictor variables selected by Lasso in a multivariable regression model using the whole sample, no variables were significant at the Bonferroni-corrected significance threshold of *p* < 0.00357 (0.05/14), but 2 variables were significant at the uncorrected threshold of *p* < 0.05. When including the 22 predictor variables selected by ENR in a multivariable regression model using the whole sample, 0 variables were significant at the Bonferroni-corrected significance threshold of *p* < 0.00227 (0.05/22), but 5 variables were significant at the uncorrected threshold of *p* < 0.05. For detailed statistics from the univariate and multivariable regression models, see Supplementary Tables S3 and S4. For a heatmap comparing the coefficient weights of all 50 predictors using a simple univariate regression and the four cross-validated machine learning models, see Supplementary Figure S4.

### 3.3. Post Hoc Analyses

#### Early Extinction

The univariate regression analysis of the Multiscale Dissociation Inventory (MDI) found an association between the MDI total score and early extinction that was significant at the *p* < 0.05 level, but would not have survived correction for multiple comparisons (B = 0.012, *p* = 0.03081). Across the six univariate regression analyses examining the six MDI subscales, two were significant at the *p* < 0.05 level, but not at the multiple comparison threshold: MDI Depersonalization (B = 0.051, *p* = 0.02486) and MDI Disengagement (B = 0.056, *p* = 0.01914).

Across the four univariate regression analyses examining the four PCL-5 PTSD symptom cluster scores, only cluster E was significant (B = 0.052, *p* = 0.00692). This finding survived correction for multiple comparisons across the four symptom clusters (0.05/4 = 0.01250), but would not have survived correction across all the univariate regressions used to examine early extinction.

## 4. Discussion

Identification of the statistical modeling approaches and patient characteristics that predict fear extinction in PTSD may eventually inform advances in precision extinction-based treatments [3,15]. Building on prior evidence that penalized regression modeling may increase the predictive accuracy in clinical research [11,65,67], we compared the accuracy of fear extinction predictions from three types of penalized regressions and traditional OLS regression in a cross-validated training sample and holdout sample. In line with our first hypothesis, all three penalized regression models were more accurate than the OLS regression in both samples. In line with our second hypothesis, the startle variables were more likely to be selected as predictors relative to the non-startle variables. Exploratory comparisons between the patient characteristics highlight three consistent predictors of early extinction: dark-enhanced startle, trait depersonalization/derealization, and PTSD hyperarousal symptom severity. Overall, our study yields novel insights into which modeling approaches and patient characteristics may reliably predict fear extinction in PTSD.

### 4.1. Modeling Approaches

The model comparisons indicated that the penalized regressions predicted fear extinction with a greater accuracy than the conventional (OLS) regression. Based on the MSEs for the models predicting early extinction, the OLS regression had 85% more predictive error than the least accurate penalized regression in the cross-validated training sample (2454.13 − 1325.76 = 1128.37; 1128.37/1325.76 = 0.851111 × 100 = 85.1111%). In the holdout sample, the OLS regression had 394% more predictive error than the least accurate penalized regression. Similarly, for the models predicting late extinction, the level of error for the OLS regression was more than double that for the least accurate penalized regression in both samples. In summary, all three penalized regression models were substantially more accurate and more generalizable to a holdout sample than the conventional regression. In contrast, the difference in the MSEs between the most and least accurate penalized regression models was within 4% in both samples and during both phases. Thus, the three penalized regressions had relatively comparable predictive performances [106]. Overall, these results suggest that penalized regressions may hold promise for helping to develop clinically useful predictions of exposure therapy responses in PTSD. However, treatment studies are needed to test this theory directly.

### 4.2. Predictor Variables

Our study is the first to demonstrate that adults with PTSD symptoms who exhibit heightened, *unconditioned* fear in an anxiety-inducing context also display deficient *conditioned* fear extinction learning. Across the 50 variables examined during early extinction, dark-enhanced startle was the only variable identified as a predictor across all eight criteria in our study. Notably, the effect of dark-enhanced startle on early extinction was significant in the multivariable models that controlled for baseline startle and the FPS during *Acquisition*, suggesting that it has an effect above-and-beyond individual differences in general startle reactivity and conditioned fear before starting extinction learning. One possible explanation is that early extinction in an uninstructed paradigm like ours, where participants are not explicitly told that the CS+ will not be followed by the US during extinction, has been found to partially capture a participant’s response to an uncertain threat [107]. Because dark-enhanced startle falls under the RDoC construct of a potential threat (“anxiety”) [108,109,110], our findings may suggest that individual differences in response to these potential threats partially modulate early extinction. Although fear conditioning falls under the RDoC construct of an acute threat (“fear”) [109,111], it has been proposed that the RDoC domains of potential and acute threats conceptually overlap within a higher-order internalizing dimension [112]. Our finding that dark-enhanced startle consistently and robustly predicted early extinction aligns with this theory. Importantly, prior treatment studies have found that *conditioned* physiological responses to trauma-related threats could be valuable for developing personalized exposure therapies for trauma-induced psychopathology [3,5]. Our finding suggests that an elevated startle in an *unconditioned* anxiety-inducing context, measured before treatment, may have additional utility in identifying trauma-exposed patients who are likely to have difficulty extinguishing fear during exposure therapy. However, clinical treatment studies are needed to test this hypothesis directly.

Our study is also the first to show that trauma-exposed individuals with elevated dissociation, specifically depersonalization and derealization, may experience deficient fear extinction learning. Among the 50 variables examined during early extinction, the depersonalization and derealization subscale of the Dissociative Experiences Scale emerged as one of the two non-startle variables predicting extinction across all the modeling approaches. Our finding that the depersonalization subscale of the MDI was also associated with early extinction increases the confidence of our findings. It extends it to a clinical measure of dissociation that has previously been found to be relevant to PTSD treatment [113], physiology [37,114,115], and clinical presentation [116]. Elevated dissociation has been theorized to hinder safety learning in PTSD, leading to heightened fear responses to nonthreatening stimuli [37]. Our study supports this theory, suggesting that individuals prone to dissociation may be less attentive during early extinction, and therefore more likely to experience a delay in learning that the CS+ no longer signals danger. Additionally, we found that the disengagement subscale of the MDI was also associated with an elevated FPS during early extinction in the univariate regression. However, it is important to note that these univariate and multivariate effects did not survive the Bonferroni correction, emphasizing the need for replication in larger samples.

Our finding that PTSD hyperarousal symptoms predicted the FPS during early extinction partially aligns with the prior literature and may have clinical implications. Prior evidence has indicated that PTSD patients with elevated arousal-related symptoms may benefit from tailored treatment approaches designed to address these specific symptoms (for review, see [117]). Therefore, our finding suggests that targeted treatments for PTSD patients with elevated hyperarousal should account for the possibility of delayed or deficient fear extinction. In line with this finding, a previous study by Galatzer-Levy et al. (2017) found that a statistically identified latent subgroup of trauma-exposed adults who had elevated FPSs to a CS+ during early extinction also had elevated DSM-IV hyperarousal symptoms [18]. Similarly, Richards et al. (2022) found that a higher FPS across both conditioned stimuli (CS+ and CS− combined) during early extinction was correlated with elevated DSM-IV hyperarousal symptoms [20]. However, two prior studies found that DSM-IV intrusion, but not hyperarousal symptoms, were associated with the FPS to a CS+ during early extinction [16,19], contrasting with our findings. A post hoc analysis of our data found that the association of early extinction with PTSD hyperarousal symptoms (and no other symptom clusters) was consistent across two measures of PTSD symptoms, suggesting that these divergent findings may stem from sample heterogeneity, rather than measurement differences (see the Supplementary Materials for additional details).

### 4.3. Methodological Considerations

Our findings suggest that it may be more challenging to identify the modeling approaches and clinical characteristics that robustly predict late extinction relative to early extinction. Overall, our machine learning prediction models had a worse accuracy and generalizability for late extinction. Although the MSE and MAE could not be compared across the different outcome variables, the coefficient of determination (R^2^) provided a standardized measure of the model performance relative to chance [106]. A comparison of the R^2^ values across the penalized regression models suggests that the predictions were more precise in the training sample for early extinction (R^2^ range 0.48–0.50) versus late extinction (R^2^ range 0.28–0.29). Similarly, in the holdout sample, the R^2^ values were higher for early extinction (R^2^ range 0.53–0.57) versus late extinction (R^2^ range 0.25–0.29). A similar pattern extended to the OLS models (see Table 3 and Table 4). Additionally, there were fewer consistent predictor variables for late extinction compared to early extinction. For example, across all the 50 variables tested, the average number of the predictor criteria met was 1.46 for early extinction versus 1.12 for late extinction. When excluding the startle variables, this difference increased, with the average number of the predictor criteria being 1.12 for early extinction versus 0.5 for late extinction. This aligns with previous research, indicating that the relationship between fear extinction and clinical variables is influenced by the temporal dynamics and operationalization of fear extinction [9,18]. Therefore, future studies focusing on clinical correlates and predictors of fear extinction may be more likely to find an effect during early extinction, where there is generally a greater variability.

Although intuitive, the higher likelihood of finding predictors when using startle versus non-startle variables underscores two critical challenges for FPS studies of fear extinction: (1) the importance of controlling for differences in general startle reactivity [57], and (2) the difficulty in identifying consistent relationships across different measurement methods [6]. For both early and late extinction, the startle variables met an average number of 10 predictor criteria, while the non-startle variables (clinical, self-reported, and demographic variables) met only 0.81 criteria. Across the 13 non-startle physiological variables (i.e., heart rate, HRV, and SCR variables), none met more than 2 out of the 8 predictor criteria, indicating a lack of consistent physiological predictors that were not startle variables. In contrast, the Lasso and ENR models for early and late extinction did retain multiple non-startle physiological measures as predictors. Further, each modality of psychophysiological measure examined (heart rate, HRV, and SCR) was retained in at least one cross-validated machine learning model, suggesting that these measures may still have contributed meaningfully to the variance in the FPS during extinction. Overall, these observations are consistent with prior evidence that individual differences in fear extinction may result from the combined effects of numerous individual difference variables, with small but meaningful individual impacts [6]. Furthermore, the finding that most candidate predictors were only identified using a subset of criteria and modeling approaches contributes to the growing evidence that methodological differences can lead to inconsistent findings in fear extinction research [9,23,118,119].

### 4.4. Limitations and Future Directions

Our study’s limitations need to be considered when interpreting its results. Statistical power has been a concern in psychophysiology research, especially when investigating individual differences and conducting multiple comparisons [120,121]. Our study was not well-powered for detecting small effects that may be reflected in the broader PTSD population. As a result, although most variables examined in our study were not consistent predictors of extinction, this does not mean that a consistent effect would not be found in a larger sample. Thus, the small sample size limits the generalizability of the findings, which will require replication in larger samples. Moreover, our sample was predominantly white and female, limiting the generalizability of our findings to samples with different racial and sex compositions. To address this limitation, replication with more diverse samples remains an important future direction. Further, our limited statistical power precluded us from examining modeling approaches that account for interaction effects [106]. Because the relationships between fear extinction and many of the variables in our study are likely to be complex and interactive [6], we propose that future studies with larger samples should build on this work. For example, future studies may expand upon the methodological framework employed in this study by including interaction effects within a penalized regression framework and by exploring machine learning approaches, such as decision trees, designed to identify these interactions. Furthermore, it is worth noting that, while FPS is a promising translational measure of conditioned fear in PTSD samples [72], it indexes only one facet of the fear response [73]. The variables that were not consistent predictors of the FPS during extinction in our study may be consistent predictors of other fear extinction measures, such as amygdala activity or subjective fear [73]. Thus, we propose that future studies extend this work by applying penalized regressions with supplemental univariate and multivariable regressions to identify the consistent predictors of other fear extinction measures.

## 5. Conclusions

In summary, we conducted a series of cross-validated penalized regressions, cross-validated OLS regressions, and multivariable- and univariate-regression-based significance tests to identify the modeling approaches and participant characteristics that predict fear extinction in traumatized adults with a continuum of PTSD symptoms. The penalized regressions outperformed the conventional OLS regression during both the early and late extinction of the FPS, as demonstrated in the training and holdout samples. We identified two novel predictors of early extinction: dark-enhanced startle and trait depersonalization/derealization. Additionally, we extended the previous findings that arousal-related PTSD symptom severity may predict early extinction. Future studies are needed to replicate and extend these findings, particularly regarding their clinical implications. Despite its limitations, our study demonstrates the effectiveness of penalized regressions and offers valuable insights into predicting fear extinction in PTSD samples. In time, this line of work may inform the development of precision extinction therapies for individuals with post-traumatic stress.

## Figures and Tables

**Table 1:** Sample demographic and clinical characteristics.

Variable	Mean ± SD or N (%)	Missing (%)
Age, years	29.48 ± 8.27	0 (0)
Sex, female	96 (77)	0 (0)
Race/Ethnicity		0 (0)
Asian	13 (10)	
Black	10 (8)	
Hispanic non-white	12 (10)	
Other	2 (2)	
white	88 (70)	
CAPS Total	29.10 ± 10.59	0 (0)
Intrusion (Cluster B)	7.79 ± 3.14	0 (0)
Avoidance (Cluster C)	3.48 ± 1.75	0 (0)
Negative Cognitions (Cluster D)	10.73 ± 5.15	0 (0)
Hyperarousal (Cluster E)	7.47 ± 3.72	0 (0)
PCL-5 Total	39.73 ± 13.65	0 (0)
CD-RISC Total	25.01 ± 6.99	3 (2.4)
STAI Trait Total	49.56 ± 10.95	4 (3.2)
STAI State Total	38.77 ± 10.35	4 (3.2)
SHAPS Total	27.73 ± 7.05	3 (2.4)
PSQI Total	8.19 ± 3.78	5 (4)
LEC Experienced + Witnessed	7.36 ± 4.06	0
LEC Experienced	4.48 ± 2.03	0
CTQ Total	58.44 ± 19.44	2 (1.6)
Sexual Abuse	10.89 ± 6.41	1 (0.8)
Physical Neglect	9.25 ± 4.07	1 (0.8)
Physical Abuse	9.41 ± 4.94	2 (1.6)
Emotional Neglect	14.41 ± 5.217	1 (0.8)
Emotional Abuse	14.51 ± 5.73	2 (1.6)
FSS Total	162.66 ± 38.36	3 (2.4)
ASI Total	25.56 ± 12.56	2 (1.6)
BDI Total	21.16 ± 10.14	0 (0)
DES Total	17.23 ± 10.72	3 (2.4)
Depersonalization and Derealization	10.04 ± 13.53	3 (2.4)
Amnesia and Dissociation	7.41 ± 7.22	3 (2.4)
DES Absorption and Imagination	25.51 ± 15.2	3 (2.4)

Note. CAPS = Clinician-Administered PTSD Scale, LEC = Life Events Checklist, FSS = Fear Survey Schedule, ASI = Anxiety Sensitivity Index, CD-RISC = Connor–Davidson Resilience Scale, CTQ = Childhood Trauma Questionnaire, PCL-5 = PTSD Checklist for DSM-5, DES = Dissociative Experiences Scale, BDI = Beck Depression Inventory II, PSQI = Pittsburgh Sleep Quality Index, SHAPS = Snaith–Hamilton Anhedonia Pleasure Scale, and STAI = State Trait Anxiety Inventory.

**Table 2:** Candidate predictors and outcome measures examined in this study, including estimates of internal consistency for multi-item scales and psychophysiological measures.

Predictors	
Clinical Measures	Demographics
CAPS for DSM-5 total score (α = 0.87)	Age ^^
CAPS Intrusion (Cluster B) score (α = 0.68) ^^	Race (white, non-Hispanic) *^
CAPS Avoidance (Cluster C) score (α = 0.61) **^^^^	Sex (Female) ***
CAPS Negative Cognitions (Cluster D) score (α = 0.72)	
CAPS Hyperarousal (Cluster E) score (α = 0.63) *****^^	
**Self-Report Measures**	**Psychophysiological Measures**
PTSD Checklist for DSM-5 Total Score (α = 0.90) **	Baseline Startle (r_SB_ = 0.96) ****^^^^^^
LEC. Experienced + Witnessed (α = 0.76) *^^	Dark-Enhanced Startle (r_SB_ = 0.74) ********^^^
LEC Experienced (α = 0.62)	FPS to CS+, Acq block 1 (r_SB_ = 0.79) *****
CTQ total score (α = 0.86) ^	FPS to CS+, Acq block 2 (r_SB_ = 0.81) ****^^^^^
CTQ Emotional Abuse subscale score (α = 0.89) *	FPS to CS+, Acq block 3 (r_SB_ = 0.75) *****^^
CTQ Emotional Neglect subscale score (α = 0.92) *^^	FPS to CS−, Acq block 1 (r_SB_ = 0.71) *^^^
CTQ Physical Abuse subscale score (α = 0.87)	FPS to CS−, Acq block 2 (r_SB_ = 0.78) ****^^^
CTQ Physical Neglect subscale score (α = 0.80) ***^^^	FPS to CS−, Acq block 3 (r_SB_ = 0.67) ****^^^^^
CTQ Sexual Abuse subscale score (α = 0.92)	HR, Baseline (r_SB_ = 0.98)
DES total score (α = 0.90) **	HR, Dark-Enhanced Startle (r_SB_ = 0.98) *
DES Absorption subscale score (α = 0.81) *	HR, Dark blocks Dark-Enhanced Startle (r_SB_ = 0.98)
DES Amnesia subscale score (α = 0.67)	HR, Light blocks Dark-Enhanced Startle (r_SB_ = 0.98)
DES Depersonalization subscale score (α = 0.82) *****	HR, Acq (r_SB_ = 0.99) *^
Anxiety Sensitivity Index total score (α = 0.88) **	HRV, Baseline (r_SB_ = 0.94) **
STAI, State Anxiety score (α = 0.93)	HRV, Dark-Enhanced Startle (r_SB_ = 0.96) ^^
STAI, Trait Anxiety score (α = 0.92) *	HRV, Dark blocks Dark-Enhanced Startle (r_SB_ = 0.92)
BDI total score (α = 0.89) *^^	HRV, Light blocks Dark-Enhanced Startle (r_SB_ = 0.91)
PSQI total score (α = 0.73) ***^^	HRV, Acq (r_SB_ = 0.99)
CDRISC total score (α = 0.85)	SCR Difference score, Acq block 1 (r_SB_ = 0.71)
Fear Survey Schedule total score (α = 0.93) ^	SCR Difference score, Acq block 2 (r_SB_ = 0.65) ^^
Snaith–Hamilton Pleasure Scale total score (α = 0.89)	SCR Difference score, Acq block 3 (r_SB_ = 0.58)
**Outcome Measures**	
FPS to CS+, Early Extinction (r_SB_ = 0.82)	FPS to CS+, Late Extinction (r_SB_ = 0.73)

Note. Each * indicates one predictor criterion (as defined in the “Examining Specific Predictor Variables” Subsection of the Section 2) that was met for early extinction; Each ^ indicates one predictor criterion (as defined in the “Examining Specific Predictor Variables” Subsection of the Section 2) that was met for late extinction; α = Cronbach’s alpha; r_SB_ = Spearman–Brown corrected split-half internal consistency; CAPS = Clinician-Administered PTSD Scale for DMS-5; LEC = Life Events Checklist; CTQ: Childhood Trauma Questionnaire; DES = Dissociative Experiences Scale; FPS = Fear-Potentiated Startle; HR = Heart Rate HRV = Heart Rate Variability; Acq = Acquisition; SCR = Skin Conductance Response; STAI = State and Trait Anxiety Index; BDI = Beck Depression Inventory, II; PSQI = Pittsburgh Sleep Quality Index; and CDRISC = Connor–Davidson resilience Scale.

**Table 3:** Performance of algorithms predicting early extinction in the (A) cross-validated training sample, and (B) holdout sample.

**(** **A) Cross-Validated Training Sample**			
**Algorithm**	**MSE**	**MAE**	**R^2^**
OLS Linear Regression	2454.13	39.08	0.32
Ridge Regression	1301.99	28.02	0.49
Lasso Regression	1325.76	28.31	0.48
Elastic Net Regression	1296.41	28.30	0.50
**(B) Holdout Sample**			
**Algorithm**	**MSE**	**MAE**	**R^2^**
OLS Linear Regression	3947.74	49.80	−0.29
Ridge Regression	799.81	22.50	0.53
Lasso Regression	794.48	21.92	0.54
Elastic Net Regression	726.91	21.29	0.57

Note. MSE = mean square error; MAE = mean absolute error; R^2^ = 1 − (MSE/var(y)); and OLS = ordinary least squares.

**Table 4:** Performance of cross-validated algorithms predicting late extinction in the (A) cross-validated training, and (B) the holdout sample.

**(A) Cross-Validated Training Sample**			
**Algorithm**	**MSE**	**MAE**	**R^2^**
OLS Linear Regression	2114.49	37.74	0.14
Ridge Regression	987.29	24.05	0.29
Lasso Regression	1000.88	24.82	0.28
Elastic Net Regression	966.55	23.60	0.29
**(B) Holdout Sample**			
**Algorithm**	**MSE**	**MAE**	**R^2^**
OLS Linear Regression	4747.17	48.26	−0.61
Ridge Regression	2153.86	32.49	0.25
Lasso Regression	2037.19	32.18	0.29
Elastic Net Regression	2087.37	32.33	0.28

Note. MSE = mean square error; MAE = mean absolute error; R^2^ = 1 − (MSE/var(y)); and OLS = ordinary least squares.

## Data Availability

Data and analysis code are available at https://osf.io/jzmcg/, accessed on 13 June 2023.

## Supplementary Materials

The following supporting information can be downloaded at: https://www.mdpi.com/article/10.3390/brainsci13081131/s1, Supplementary Background provides a more detailed literature review on factors predicting fear extinction. Supplementary Methods provide more details regarding clinical and self-report measures included in this study. Supplementary Tables show beta weights and *p*-values from all univariate regression models. Supplementary Figures display the distribution of FPS during early extinction and late extinction in the full, training, and holdout samples. Supplementary Discussion provides more detailed interpretation of post hoc analyses of PTSD symptoms using the PCL-5. Supplementary References that appear in the supplement in addition to those in the manuscript [122,123,124,125,126,127,128,129,130,131,132,133,134,135,136,137,138,139,140,141,142,143,144,145,146,147,148,149,150,151,152,153,154,155,156,157,158,159,160,161,162,163,164].

## Appendix A

**Table A1:** Candidate predictors that met at least one criterion for early extinction.

Variable	UN BF	UN 0.05	Lasso	ENR	MV Lasso BF	MV Lasso 0.05	MV ENR BF	MV ENR 0.05
CAPS Avoidance			X	X				
CAPS Hyperarousal		X	X	X		X		X
White, Non-Hispanic				X				
Sex (Female)		X	X	X				
PCL Total				X				X
LEC Experienced + Witnessed				X				
CTQ Emotional Abuse				X				
CTQ Emotional Neglect				X				
CTQ Physical Neglect			X	X				X
DES Total		X		X				
DES Absorption		X						
DES Depersonalization		X	X	X		X		X
ASI Total			X	X				
STAI Trait Total		X						
BDI Total		X						
PSQI Total		X	X	X				
Baseline Startle	X	X		X				X
Dark-Enhanced Startle	X	X	X	X	X	X	X	X
FPS to CS+, Acq block 1	X	X	X	X				X
FPS to CS+, Acq block 2	X	X	X	X				
FPS to CS+, Acq block 3	X	X	X	X		X		
FPS to CS−, Acq block 1		X						
FPS to CS−, Acq block 2	X	X	X	X				
FPS to CS−, Acq block 3	X	X	X	X				
HR, Dark-Enhanced Startle				X				
HR, Acq				X				
RSA, Baseline			X	X				

Note. BF = Bonferroni significant; 0.05 = Nominally significant at *p* < 0.05; UN = univariate; MV = multivariate; ENR = Elastic Net Ridge; CAPS-5 = Clinician-Administered PTSD Scale for DMS-5; LEC = Life Events Checklist; CTQ = Childhood Trauma Questionnaire; DES = Dissociative Experiences Scale; FPS = Fear-Potentiated Startle; HR = Heart Rate; RSA = Respiratory Sinus Arrhythmia; ASI = Anxiety Sensitivity Index; STAI = State-Trait Anxiety Inventory; BDI = Beck Depression Inventory; PSQI = Pittsburgh Sleep Quality Index; and Acq = Acquisition. The *p*-value significance thresholds were as follows: BF UN = *p* < 0.001; UN 0.05 = *p* < 0.05; MV Lasso BF = *p* < 0.004; MV Lasso 0.05 = *p* < 0.05; MV ENR BF = *p* < 0.002; and MV ENR 0.05 = *p* < 0.05.

**Table A2:** Candidate predictors that met at least one criterion for late extinction.

Variable	UN BF	UN 0.05	Lasso	ENR	MV Lasso BF	MV Lasso 0.05	MV ENR BF	MV ENR 0.05
CAPS Intrusion			X	X				
CAPS Avoidance		X	X	X				X
CAPS Hyperarousal				X				X
Age				X				X
White, Non-Hispanic				X				
LEC Experienced + Witnessed			X	X				
CTQ total score				X				
CTQ Emotional Neglect			X	X				
CTQ Physical Neglect		X	X	X				
BDI Total			X	X				
PSQI Total			X	X				
FSS Total				X				
Baseline Startle	X	X	X	X		X		X
Dark-Enhanced Startle		X	X	X				
FPS to CS+, Acq block 2	X	X	X	X		X		
FPS to CS+, Acq block 3		X		X				
FPS to CS−, Acq block 1		X	X	X				
FPS to CS−, Acq block 2	X	X		X				
FPS to CS−, Acq block 3	X	X	X	X				X
Heart Rate, Acq				X				
RSA, Dark-Enhanced Startle			X	X				
SCR Difference score (CS+-CS−) Acq block 2			X	X				

Note. BF = Bonferroni significant; 0.05 = Nominally significant at *p* < 0.05; UN = univariate; MV = multivariate; ENR = Elastic Net Ridge; CAPS-5 = Clinician-Administered PTSD Scale for DMS-5; LEC = Life Events Checklist; CTQ = Childhood Trauma Questionnaire; DES = Dissociative Experiences Scale; FPS = Fear-Potentiated Startle; HR = Heart Rate; RSA = Respiratory Sinus Arrhythmia; ASI = Anxiety Sensitivity Index; STAI = State-Trait Anxiety Inventory; BDI = Beck Depression Inventory; PSQI = Pittsburgh Sleep Quality Index; and Acq = Acquisition. The *p*-value significance thresholds were as follows: BF UN = *p* < 0.001; UN 0.05 = *p* < 0.05; MV Lasso BF = *p* < 0.004; MV Lasso 0.05 = *p* < 0.05; MV ENR BF = *p* < 0.002; and MV ENR 0.05 = *p* < 0.05.

## References

[1] Ressler K.J., Berretta S., Bolshakov V.Y., Rosso I.M., Meloni E.G., Rauch S.L., Carlezon W.A. (2022). Post-traumatic stress disorder: Clinical and translational neuroscience from cells to circuits. Nat. Rev. Neuro..

[2] Rauch S.A.M., Eftekhari A., Ruzek J.I. (2012). Review of exposure therapy: A gold standard for PTSD treatment. J. Rehabil. Res. Dev..

[3] Norrholm S.D., Jovanovic T., Gerardi M., Breazeale K.G., Price M., Davis M., Duncan E., Ressler K.J., Bradley B., Rizzo A. (2016). Baseline psychophysiological and cortisol reactivity as a predictor of PTSD treatment outcome in virtual reality exposure therapy. Behav. Res. Ther..

[4] Kirlic N., Kuplicki R., Touthang J., Cohen Z.P., Stewart J.L., Paulus M.P., Aupperle R.L. (2022). Behavioral and neural responses during fear conditioning and extinction in a large transdiagnostic sample. NeuroImage Clin..

[5] Wangelin B.C., Tuerk P.W. (2015). Taking the pulse of prolonged exposure therapy: Physiological reactivity to trauma imagery as an objective measure of treatment response. Depress. Anxiety.

[6] Lonsdorf T.B., Merz C.J. (2017). More than just noise: Inter-individual differences in fear acquisition, extinction and return of fear in humans—Biological, experiential, temperamental factors, and methodological pitfalls. Neurosci. Biobehav. Rev..

[7] Pöhlchen D., Leuchs L., Binder F.P., Blaskovich B., Nantawisarakul T., Topalidis P., Brückl T.M., Norrholm S.D., Jovanovic T., BeCOME Working Group (2020). No robust differences in fear conditioning between patients with fear-related disorders and healthy controls. Behav. Res. Ther..

[8] Wen Z., Fried J., Pace-Schott E.F., Lazar S.W., Milad M.R. (2022). Revisiting sex differences in the acquisition and extinction of threat conditioning in humans. Learn. Mem..

[9] Lewis M.W., Bradford D.E., Pace-Schott E.F., Rauch S.L., Rosso I.M. (2023). Multiverse analyses of fear acquisition and extinction retention in posttraumatic stress disorder. Psychophysiology.

[10] Sjouwerman R., Scharfenort R., Lonsdorf T.B. (2020). Individual differences in fear acquisition: Multivariate analyses of different emotional negativity scales, physiological responding, subjective measures, and neural activation. Sci. Rep..

[11] Webb C.A., Cohen Z.D., Beard C., Forgeard M., Peckham A.D., Björgvinsson T. (2020). Personalized prognostic prediction of treatment outcome for depressed patients in a naturalistic psychiatric hospital setting: A comparison of machine learning approaches. J. Consult. Clin. Psychol..

[12] Greenwood C.J., Youssef G.J., Letcher P., Macdonald J.A., Hagg L.J., Sanson A., Mcintosh J., Hutchinson D.M., Toumbourou J.W., Fuller-Tyszkiewicz M. (2020). A comparison of penalised regression methods for informing the selection of predictive markers. PLoS ONE.

[13] Studerus E., Vizeli P., Harder S., Ley L., Liechti M.E. (2021). Prediction of MDMA response in healthy humans: A pooled analysis of placebo-controlled studies. J. Psychopharmacol..

[14] van Rooij S.J.H., Stevens J.S., Ely T.D., Hinrichs R., Michopoulos V., Winters S.J., Ogbonmwan Y.E., Shin J., Nugent N.R., Hudak L.A. (2018). The role of the hippocampus in predicting future posttraumatic stress disorder symptoms in recently traumatized civilians. Biol. Psychiatry.

[15] Zuj D.V., Palmer M.A., Lommen M.J.J., Felmingham K.L. (2016). The centrality of fear extinction in linking risk factors to PTSD: A narrative review. Neurosci. Biobehav. Rev..

[16] Norrholm S.D., Jovanovic T., Olin I.W., Sands L.A., Karapanou I., Bradley B., Ressler K.J. (2011). Fear extinction in traumatized civilians with posttraumatic stress disorder: Relation to symptom severity. Biol. Psychiatry.

[17] Orcutt H.K., Hannan S.M., Seligowski A.V., Jovanovic T., Norrholm S.D., Ressler K.J., McCanne T. (2016). Fear-potentiated startle and fear extinction in a sample of undergraduate women exposed to a campus mass shooting. Front. Psychol..

[18] Galatzer-Levy I.R., Andero R., Sawamura T., Jovanovic T., Papini S., Ressler K.J., Norrholm S.D. (2017). A cross species study of heterogeneity in fear extinction learning in relation to FKBP5 variation and expression: Implications for the acute treatment of posttraumatic stress disorder. Neuropharmacology.

[19] Norrholm S.D., Glover E.M., Stevens J.S., Fani N., Galatzer-Levy I.R., Bradley B., Ressler K.J., Jovanovic T. (2015). Fear load: The psychophysiological over-expression of fear as an intermediate phenotype associated with trauma reactions. Int. J. Psychophysiol..

[20] Richards A., Inslicht S.S., Yack L.M., Metzler T.J., Russell Huie J., Straus L.D., Dukes C., Hubachek S.Q., Felmingham K.L., Mathalon D.H. (2022). The relationship of fear-potentiated startle and polysomnography-measured sleep in trauma-exposed men and women with and without PTSD: Testing REM sleep effects and exploring the roles of an integrative measure of sleep, PTSD symptoms, and biological Sex. Sleep.

[21] Galatzer-Levy I.R., Bryant R.A. (2013). 636,120 Ways to have posttraumatic stress disorder. Perspect. Psychol. Sci..

[22] Zoellner L.A., Pruitt L.D., Farach F.J., Jun J.J. (2014). Understanding heterogeneity in PTSD: Fear, dysphoria, and distress. Depress. Anxiety.

[23] Lonsdorf T.B., Gerlicher A., Klingelhöfer-Jens M., Krypotos A.-M. (2022). Multiverse analyses in fear conditioning research. Behav. Res. Ther..

[24] Pappens M., Schroijen M., Sütterlin S., Smets E., den Bergh O.V., Thayer J.F., Diest I.V. (2014). Resting heart rate variability predicts safety learning and fear extinction in an interoceptive fear conditioning paradigm. PLoS ONE.

[25] Bottary R., Seo J., Daffre C., Gazecki S., Moore K.N., Kopotiyenko K., Dominguez J.P., Gannon K., Lasko N.B., Roth B. (2020). Fear extinction memory is negatively associated with REM sleep in insomnia disorder. Sleep.

[26] Pace-Schott E.F., Germain A., Milad M.R. (2015). Effects of sleep on memory for conditioned fear and fear extinction. Psychol. Bull..

[27] Pace-Schott E.F., Seo J., Bottary R. (2023). The influence of sleep on fear extinction in trauma-related disorders. Neurobiol. Stress.

[28] Schenker M.T., Ince S., Ney L.J., Hsu C.-M.K., Zuj D.V., Jordan A.S., Nicholas C.L., Felmingham K.L. (2022). Sex differences in the effect of subjective sleep on fear conditioning, extinction learning, and extinction recall in individuals with a range of PTSD symptom severity. Behav. Res. Ther..

[29] Marusak H.A., Hehr A., Bhogal A., Peters C., Iadipaolo A., Rabinak C.A. (2021). Alterations in fear extinction neural circuitry and fear-related behavior linked to trauma exposure in children. Behav. Brain. Res..

[30] Jenness J.L., Miller A.B., Rosen M.L., McLaughlin K.A. (2019). Extinction learning as a potential mechanism linking high vagal tone with lower PTSD symptoms among abused youth. J. Abnorm. Child Psychol..

[31] Hermann A., Küpper Y., Schmitz A., Walter B., Vaitl D., Hennig J., Stark R., Tabbert K. (2012). Functional gene polymorphisms in the serotonin system and traumatic life events modulate the neural basis of fear acquisition and extinction. PLoS ONE.

[32] Kuhn M., Höger N., Feige B., Blechert J., Normann C., Nissen C. (2014). Fear extinction as a model for synaptic plasticity in major depressive disorder. PLoS ONE.

[33] Rainer C., Nasrouei S., Tschofen S., Bliem H.R., Wilhelm F.H., Marksteiner J. (2020). Fear acquisition and extinction in elderly patients with depression. J. Affect. Disord..

[34] Wendt J., Neubert J., Koenig J., Thayer J.F., Hamm A.O. (2015). Resting heart rate variability is associated with inhibition of conditioned fear. Psychophysiology.

[35] Seligowski A.V., Lee D.J., Miron L.R., Orcutt H.K., Jovanovic T., Norrholm S.D. (2016). Prospective associations between emotion dysregulation and fear-potentiated startle: The moderating effect of respiratory sinus arrhythmia. Front. Psychol..

[36] Giesbrecht T., Smeets T., Merckelbach H., Jelicic M. (2007). Depersonalization experiences in undergraduates are related to heightened stress cortisol responses. J. Nerv. Ment. Dis..

[37] Seligowski A.V., Lebois L.A.M., Hill S.B., Kahhale I., Wolff J.D., Jovanovic T., Winternitz S.R., Kaufman M.L., Ressler K.J. (2019). Autonomic responses to fear conditioning among women with PTSD and dissociation. Depress. Anxiety.

[38] van Rooij S.J.H., Ravi M., Ely T.D., Michopoulos V., Winters S.J., Shin J., Marin M.-F., Milad M.R., Rothbaum B.O., Ressler K.J. (2021). Hippocampal activation during contextual fear inhibition related to resilience in the early aftermath of trauma. Behav. Brain Res..

[39] Rakesh G., Morey R.A., Zannas A.S., Malik Z., Marx C.E., Clausen A.N., Kritzer M.D., Szabo S.T. (2019). Resilience as a translational endpoint in the treatment of PTSD. Mol. Psychiatry.

[40] DePierro J.M., D’Andrea W., Frewen P., Ritsner M.S. (2014). Anhedonia in trauma related disorders: The good, the bad, and the shut-down. Anhedonia: A Comprehensive Handbook Volume II: Neuropsychiatric and Physical Disorders.

[41] Olson E.A., Kaiser R.H., Pizzagalli D.A., Rauch S.L., Rosso I.M. (2018). Anhedonia in trauma-exposed individuals: Functional connectivity and decision-making correlates. Biol. Psychiatry Cogn. Neurosci. Neuroimaging.

[42] Walker R.S.W. (2017). The Function of Conditioned Fear in Reward Propensity: Evidence for Interrelated Approach-Avoid Systems. Ph.D. Thesis.

[43] Lewis M.W., Jones R.T., Davis M.T. (2020). Exploring the impact of trauma type and extent of exposure on posttraumatic alterations in 5-HT1A expression. Transl. Psychiatry.

[44] Stenson A.F., van Rooij S.J.H., Carter S.E., Powers A., Jovanovic T. (2021). A legacy of fear: Physiological evidence for intergenerational effects of trauma exposure on fear and safety signal learning among African Americans. Behav. Brain Res..

[45] Hunt C., Cooper S.E., Hartnell M.P., Lissek S. (2019). Anxiety sensitivity and intolerance of uncertainty facilitate associations between generalized Pavlovian fear and maladaptive avoidance decisions. J. Abnorm. Psychol..

[46] Lebeaut A., Tran J.K., Vujanovic A.A. (2020). Posttraumatic stress, alcohol use severity, and alcohol use motives among firefighters: The role of anxiety sensitivity. Addict. Behav..

[47] Scharff A., Ortiz S.N., Forrest L.N., Smith A.R. (2021). Comparing the clinical presentation of eating disorder patients with and without trauma history and/or comorbid PTSD. Eat. Disord..

[48] Taylor S., Koch W.J., McNally R.J. (1992). How does anxiety sensitivity vary across the anxiety disorders?. J. Anxiety Disord..

[49] Taylor S. (2003). Anxiety sensitivity and its implications for understanding and treating PTSD. J. Cogn. Psychother..

[50] Carpenter J.K., Bragdon L., Pineles S.L. (2022). Conditioned physiological reactivity and PTSD symptoms across the menstrual cycle: Anxiety sensitivity as a moderator. Psychol. Trauma Theory Res. Pract. Policy.

[51] Overstreet C., Brown E., Berenz E.C., Brown R.C., Hawn S., McDonald S., Pickett T., Danielson C.K., Thomas S., Amstadter A. (2018). Anxiety sensitivity and distress tolerance typologies and relations to posttraumatic stress disorder: A cluster analytic approach. Mil. Psychol..

[52] Vaidyanathan U., Patrick C.J., Bernat E.M. (2009). Startle reflex potentiation during aversive picture viewing as an indicator of trait fear. Psychophysiology.

[53] Kredlow A.M., Orr S.P., Otto M.W. (2018). Who is studied in de novo fear conditioning paradigms? An examination of demographic and stimulus characteristics predicting fear learning. Int. J. Psychophysiol..

[54] Morris M.C., Hellman N., Abelson J.L., Rao U. (2016). Cortisol, heart rate, and blood pressure as early markers of PTSD risk: A systematic review and meta-analysis. Clin. Psychol. Rev..

[55] Schiweck C., Piette D., Berckmans D., Claes S., Vrieze E. (2019). Heart rate and high frequency heart rate variability during stress as biomarker for clinical depression. A systematic review. Psychol. Med..

[56] Russo A.S., Parsons R.G. (2017). Acoustic startle response in rats predicts inter-individual variation in fear extinction. Neurobiol. Learn. Mem..

[57] Bradford D.E., Kaye J.T., Curtin J.J. (2014). Not just noise: Individual differences in general startle reactivity predict startle response to uncertain and certain threat. Psychophysiology.

[58] Kamkwalala A., Norrholm S.D., Poole J.M., Brown A., Donley S., Duncan E., Bradley B., Ressler K.J., Jovanovic T. (2012). Dark-enhanced startle responses and heart rate variability in a traumatized civilian sample: Putative sex-specific correlates of posttraumatic stress disorder. Psychosom. Med..

[59] Fani N., Tone E.B., Phifer J., Norrholm S.D., Bradley B., Ressler K.J., Kamkwalala A., Jovanovic T. (2012). Attention bias toward threat is associated with exaggerated fear expression and impaired extinction in PTSD. Psychol. Med..

[60] Jovanovic T. (2010). How the neurocircuitry and genetics of fear inhibition may inform our understanding of PTSD. Am. J. Psychiatry.

[61] Burger A.M., Verkuil B., Van Diest I., Van der Does W., Thayer J.F., Brosschot J.F. (2016). The effects of transcutaneous vagus nerve stimulation on conditioned fear extinction in humans. Neurobiol. Learn. Mem..

[62] Shvil E., Rusch H.L., Sullivan G.M., Neria Y. (2013). Neural, psychophysiological, and behavioral markers of fear processing in PTSD: A review of the literature. Curr. Psychiatry Rep..

[63] Friedman J., Hastie T., Tibshirani R. (2010). Regularization paths for generalized linear models via coordinate descent. J. Stat. Softw..

[64] Privé F., Aschard H., Blum M.G.B. (2019). Efficient implementation of penalized regression for genetic risk prediction. Genetics.

[65] Ambler G., Seaman S., Omar R.Z. (2012). An evaluation of penalised survival methods for developing prognostic models with rare events. Stat. Med..

[66] Li X., Liang C., Ma F. (2022). Forecasting stock market volatility with a large number of predictors: New evidence from the MS-MIDAS-LASSO model. Ann. Oper. Res..

[67] Pavlou M., Ambler G., Seaman S.R., Guttmann O., Elliott P., King M., Omar R.Z. (2015). How to develop a more accurate risk prediction model when there are few events. BMJ.

[68] Cho S., Kim K., Kim Y.J., Lee J.-K., Cho Y.S., Lee J.-Y., Han B.-G., Kim H., Ott J., Park T. (2010). Joint identification of multiple genetic variants via elastic-net variable selection in a genome-wide association analysis. Ann. Hum. Genet..

[69] Ghosh D., Zhu Y., Coffman D.L. (2015). Penalized regression procedures for variable selection in the potential outcomes framework. Stat. Med..

[70] Tibshirani R. (1997). The lasso method for variable selection in the cox model. Stat. Med..

[71] Walter S., Tiemeier H. (2009). Variable selection: Current practice in epidemiological studies. Eur. J. Epidemiol..

[72] Glover E.M., Phifer J.E., Crain D.F., Norrholm S.D., Davis M., Bradley B., Ressler K.J., Jovanovic T. (2011). Tools for translational neuroscience: PTSD is associated with heightened fear responses using acoustic startle but not skin conductance measures. Depress. Anxiety.

[73] Lonsdorf T.B., Menz M.M., Andreatta M., Fullana M.A., Golkar A., Haaker J., Heitland I., Hermann A., Kuhn M., Kruse O. (2017). Don’t fear “fear conditioning”: Methodological considerations for the design and analysis of studies on human fear acquisition, extinction, and return of fear. Neurosci. Biobehav. Rev..

[74] Weathers F.W., Bovin M.J., Lee D.J., Sloan D.M., Schnurr P.P., Kaloupek D.G., Keane T.M., Marx B.P. (2018). The Clinician-Administered PTSD Scale for DSM–5 (CAPS-5): Development and initial psychometric evaluation in military veterans. Psychol. Assess..

[75] Lecrubier Y., Sheehan D., Weiller E., Amorim P., Bonora I., Harnett Sheehan K., Janavs J., Dunbar G. (1997). The Mini International Neuropsychiatric Interview (MINI). A short diagnostic structured interview: Reliability and validity according to the CIDI. Eur. Psychiatry.

[76] Weathers F.W., Blake D.D., Schnurr P.P., Kaloupek D.G., Marx B.P., Keane T.M. (2013). The Clinician-Administered PTSD Scale for DSM-5 (CAPS-5). www.ptsd.va.gov.

[77] Bernstein D.P., Fink L., Handelsman L., Foote J. (1994). Childhood Trauma Questionnaire.

[78] Gray M.J., Litz B.T., Hsu J.L., Lombardo T.W. (2004). Psychometric properties of the Life Events Checklist. Assessment.

[79] Blevins C.A., Weathers F.W., Witte T.K. (2014). Dissociation and posttraumatic stress disorder: A latent profile analysis. J. Trauma. Stress.

[80] Carlson E.B., Putnam F.W. (1993). An update on the Dissociative Experiences Scale. Dissociation Prog. Dissociative Disord..

[81] Briere J., Weathers F.W., Runtz M. (2005). Is dissociation a multidimensional construct? Data from the Multiscale Dissociation Inventory. J. Trauma. Stress.

[82] Beck A.T., Steer R.A., Brown G. (1996). Beck Depression Inventory-II (BDI-II).

[83] Snaith R.P., Hamilton M., Morley S., Humayan A., Hargreaves D., Trigwell P. (1995). A scale for the assessment of hedonic tone the Snaith-Hamilton Pleasure Scale. Br. J. Psychiatry.

[84] Spielberger C.D. (1983). State-Trait Anxiety Inventory for Adults.

[85] Geer J.H. (1965). The development of a scale to measure fear. Behav. Res. Ther..

[86] Taylor S., Zvolensky M.J., Cox B.J., Deacon B., Heimberg R.G., Ledley D.R., Abramowitz J.S., Holaway R.M., Sandin B., Stewart S.H. (2007). Robust dimensions of anxiety sensitivity: Development and initial validation of the Anxiety Sensitivity Index-3. Psychol. Assess..

[87] Buysse D.J., Reynolds C.F., Monk T.H., Berman S.R., Kupfer D.J. (1989). The Pittsburgh Sleep Quality Index: A new instrument for psychiatric practice and research. Psychiatry Res..

[88] Campbell-Sills L., Stein M.B. (2007). Psychometric analysis and refinement of the Connor–Davidson Resilience Scale (CD-RISC): Validation of a 10-item measure of resilience. J. Trauma. Stress.

[89] Blumenthal T.D., Cuthbert B.N., Filion D.L., Hackley S., Lipp O.V., Van Boxtel A. (2005). Committee report: Guidelines for human startle eyeblink electromyographic studies. Psychophysiology.

[90] Boucsein W., Fowles D.C., Grimnes S., Ben-Shakhar G., Roth W.T., Dawson M.E., Filion D.L. (2012). Publication Recommendations for Electrodermal Measurements. Psychophysiology.

[91] Morgan E. (2016). All about ECG Part 4: Basic Artifact Correction. MindWare Technologies Support.

[92] Stekhoven D.J., Buehlmann P. (2012). MissForest-nonparametric missing value imputation for mixed-type data. Bioinform.

[93] R Core Team, R The R Project for Statistical Computing. https://www.r-project.org/.

[94] Friedman J., Hastie T., Tibshirani R., Narasimhan B., Tay K., Simon N., Qian J., Yang J. (2023). Glmnet. Lasso and Elastic-Net Regularized Generalized Linear Models.

[95] Kuhn M. (2019). The Caret Package. https://topepo.github.io/caret/.

[96] Chicco D., Warrens M.J., Jurman G. (2021). The coefficient of determination R-squared is more informative than SMAPE, MAE, MAPE, MSE and RMSE in regression analysis evaluation. Peer J. Comput. Sci..

[97] Rubin M. (2021). When to adjust alpha during multiple testing: A consideration of disjunction, conjunction, and individual testing. Synthese.

[98] Bennett C.M., Wolford G.L., Miller M.B. (2009). The principled control of false positives in neuroimaging. Soc. Cogn. Affect. Neurosci..

[99] Cramer A.O.J., van Ravenzwaaij D., Matzke D., Steingroever H., Wetzels R., Grasman R.P.P.P., Waldorp L.J., Wagenmakers E.-J. (2016). Hidden multiplicity in exploratory multiway ANOVA: Prevalence and remedies. Psychon. Bull. Rev..

[100] Streiner D.L. (2015). Best (but oft-forgotten) practices: The multiple problems of multiplicity—Whether and how to correct for many statistical tests. Am. J. Clin..

[101] Parker R.A., Weir C.J. (2020). Non-adjustment for multiple testing in multi-arm trials of distinct treatments: Rationale and justification. Clin. Trials.

[102] Wason J.M.S., Stecher L., Mander A.P. (2014). Correcting for multiple-testing in multi-arm trials: Is it necessary and is it done?. Trials.

[103] Tutzauer F. (2003). On the sensible application of familywise alpha adjustment. Hum. Commun. Res..

[104] Bender R., Lange S. (2001). Adjusting for multiple testing—When and how?. J. Clin. Epidemiol..

[105] Blevins C.A., Weathers F.W., Davis M.T., Witte T.K., Domino J.L. (2015). The Posttraumatic Stress Disorder Checklist for DSM-5 (PCL-5): Development and initial psychometric evaluation. J. Trauma. Stress.

[106] Mohri M., Rostamizadeh A., Talwalkar A. (2018). Foundations of Machine Learning.

[107] Morriss J., van Reekum C.M. (2019). I feel safe when I know: Contingency instruction promotes threat extinction in high intolerance of uncertainty individuals. Behav. Res. Ther..

[108] Potential Threat (“Anxiety”). https://www.nimh.nih.gov/research/research-funded-by-nimh/rdoc/constructs/potential-threat-anxiety.

[109] Kozak M.J., Cuthbert B.N. (2016). The NIMH Research Domain Criteria initiative: Background, issues, and pragmatics. Psychophysiology.

[110] Cuthbert B.N. (2022). Research Domain Criteria (RDoC): Progress and potential. Curr. Dir. Psychol. Sci..

[111] Acute Threat (“Fear”). https://www.nimh.nih.gov/research/research-funded-by-nimh/rdoc/constructs/acute-threat-fear.

[112] Michelini G., Palumbo I.M., DeYoung C.G., Latzman R.D., Kotov R. (2021). Linking RDoC and HiTOP: A new interface for advancing psychiatric nosology and neuroscience. Clin. Psychol. Rev..

[113] Resick P.A., Suvak M.K., Johnides B.D., Mitchell K.S., Iverson K.M. (2012). The impact of dissociation on PTSD treatment with cognitive processing therapy. Depress. Anxiety.

[114] Powers A., Dixon H.D., Conneely K., Gluck R., Munoz A., Rochat C., Mendoza H., Hartzell G., Ressler K.J., Bradley B. (2019). The differential effects of PTSD, MDD, and dissociation on CRP in trauma-exposed women. Compr. Psychiatry.

[115] Powers A., Mekawi Y., Fickenwirth M., Nugent N.R., Dixon H.D., Minton S., Kim Y.J., Gluck R., Carter S., Fani N. (2021). Emotion dysregulation and dissociation contribute to decreased heart rate variability to an acute psychosocial stressor in trauma-exposed Black women. J. Psychiatr. Res..

[116] Powers A., Cross D., Fani N., Bradley B. (2015). PTSD, emotion dysregulation, and dissociative symptoms in a highly traumatized sample. J. Psychiatr. Res..

[117] Norrholm S.D., Jovanovic T. (2010). Tailoring therapeutic strategies for treating posttraumatic stress disorder symptom clusters. Neuropsychiatr. Dis. Treat..

[118] Kuhn M., Gerlicher A.M.V., Lonsdorf T.B. (2022). Navigating the manyverse of skin conductance response quantification approaches—A direct comparison of trough-to-peak, baseline correction, and model-based approaches in Ledalab and PsPM. Psychophysiology.

[119] Sjouwerman R., Illius S., Kuhn M., Lonsdorf T.B. (2022). A data multiverse analysis investigating non-model based SCR quantification approaches. Psychophysiology.

[120] Button K.S., Ioannidis J.P.A., Mokrysz C., Nosek B.A., Flint J., Robinson E.S.J., Munafò M.R. (2013). Power failure: Why small sample size undermines the reliability of neuroscience. Nat. Rev. Neurosci..

[121] Baldwin S.A. (2017). Improving the rigor of psychophysiology research. Int. J. Psychophysiol..

[122] Vinograd M., Stout D.M., Risbrough V.B., Pizzagalli D.A. (2022). Anhedonia in posttraumatic stress disorder: Prevalence, phenotypes, and neural circuitry. Anhedonia: Preclinical, Translational, and Clinical Integration.

[123] Beauchaine T.P., Thayer J.F. (2015). Heart rate variability as a transdiagnostic biomarker of psychopathology. Int. J. Psychophysiol..

[124] Van Calster B., van Smeden M., De Cock B., Steyerberg E.W. (2020). Regression shrinkage methods for clinical prediction models do not guarantee improved performance: Simulation study. Stat. Methods Med. Res..

[125] VanderWeele T.J., Mathur M.B. (2019). Some desirable properties of the Bonferroni correction: Is the Bonferroni correction really so bad?. Am. J. Epidemiol..

[126] Lee D.J., Weathers F.W., Thompson-Hollands J., Sloan D.M., Marx B.P. (2022). Concordance in PTSD symptom change between DSM-5 versions of the Clinician-Administered PTSD Scale (CAPS-5) and PTSD Checklist (PCL-5). Psychol. Assess..

[127] Cross D., Fani N., Powers A., Bradley B. (2017). Neurobiological development in the context of childhood trauma. Clin. Psychol. Sci. Pract..

[128] Heim C., Nemeroff C.B. (2001). The role of childhood trauma in the neurobiology of mood and anxiety disorders: Preclinical and clinical Studies. Biol. Psychiatry.

[129] Herringa R.J. (2017). Trauma, PTSD, and the developing brain. Curr. Psychiatry Rep..

[130] Nemeroff C.B. (2004). Neurobiological Consequences of Childhood Trauma. J. Clin. Psychiatry.

[131] Bosch J., Mackintosh M.-A., Wells S.Y., Wickramasinghe I., Glassman L.H., Morland L.A. (2020). PTSD treatment response and quality of life in women with childhood trauma histories. Psychol. Trauma Theory Res. Pract. Policy.

[132] McLaughlin K.A., Lambert H.K. (2017). Child trauma exposure and psychopathology: Mechanisms of risk and resilience. Curr. Opin. Psychol..

[133] Charak R., de Jong J.T.V.M., Berckmoes L.H., Ndayisaba H., Reis R. (2017). Assessing the factor structure of the Childhood Trauma Questionnaire, and cumulative effect of abuse and neglect on mental health among adolescents in conflict-affected Burundi. Child Abuse Negl..

[134] Cheng Y.-C., Kuo P.-H. (2018). Reliability and factor structure of the Chinese version of Childhood Trauma Questionnaire-Short Form in patients with substance use disorder. Taiwanese J. Psychiatry.

[135] Scher C.D., Stein M.B., Asmundson G.J., McCreary D.R., Forde D.R. (2001). The Childhood Trauma Questionnaire in a community sample: Psychometric properties and normative data. J. Trauma. Stress.

[136] Spinhoven P., Penninx B.W., Hickendorff M., van Hemert A.M., Bernstein D.P., Elzinga B.M. (2014). Childhood Trauma Questionnaire: Factor structure, measurement invariance, and validity across emotional disorders. Psychol. Assess..

[137] May C.L., Wisco B.E. (2016). Defining Trauma: How level of exposure and proximity affect risk for posttraumatic stress disorder. Psychol. Trauma Theory Res. Pract. Policy.

[138] Olson E.A., Overbey T.A., Ostrand C.G., Pizzagalli D.A., Rauch S.L., Rosso I.M. (2019). Childhood maltreatment experiences are associated with altered diffusion in occipito-temporal white matter Pathways. Brain Behav..

[139] Weis C.N., Webb E.K., Stevens S.K., Larson C.L., deRoon-Cassini T.A. (2022). Scoring the Life Events Checklist: Comparison of three scoring methods. Psychol. Trauma Theory Res. Pract. Policy.

[140] Rosso I.M., Silveri M.M., Olson E.A., Eric Jensen J., Ren B. (2022). Regional specificity and clinical correlates of cortical GABA alterations in posttraumatic stress disorder. Neuropsychopharmacology.

[141] Pugach C.P., Nomamiukor F.O., Gay N.G., Wisco B.E. (2021). Temporal stability of self-reported trauma exposure on the Life Events Checklist for DSM-5. J. Trauma. Stress.

[142] Dorahy M.J., van der Hart O. (2015). DSM–5′s Posttraumatic stress disorder with dissociative symptoms: Challenges and future directions. J. Trauma Dissociation.

[143] Lanius R.A., Vermetten E., Loewenstein R.J., Brand B., Schmahl C., Bremner J.D., Spiegel D. (2010). Emotion modulation in PTSD: Clinical and neurobiological evidence for a dissociative subtype. Am. J. Psychiatry.

[144] Lanius R.A., Brand B., Vermetten E., Frewen P.A., Spiegel D. (2012). The dissociative subtype of posttraumatic stress disorder: Rationale, clinical and neurobiological evidence, and implications. Depress. Anxiety.

[145] Schiavone F.L., Frewen P., McKinnon M., Lanius R.A. (2018). The dissociative subtype of PTSD: An update of the literature. PTSD Research Quarterly..

[146] Lyssenko L., Schmahl C., Bockhacker L., Vonderlin R., Bohus M., Kleindienst N. (2018). Dissociation in psychiatric disorders: A meta-analysis of studies using the Dissociative Experiences Scale. Am. J. Psychiatry.

[147] Carlson E.B., Rosser-Hogan R. (1991). Trauma experiences, Posttraumatic Stress, dissociation, and depression in Cambodian refugees. Am. J. Psychiatry.

[148] Ross C.A., Ellason J.W., Anderson G. (1995). A factor analysis of the Dissociative Experiences Scale (DES) in dissociative identity disorder. Dissociation Prog. Dissociative Disord..

[149] Stockdale G.D., Gridley B.E., Balogh D.W., Holtgraves T. (2002). Confirmatory factor analysis of single- and multiple-factor competing models of the dissociative experiences scale in a nonclinical sample. Assessment.

[150] Patel H., O’Connor C., Andrews K., Amlung M., Lanius R., McKinnon M.C. (2022). Dissociative symptomatology mediates the relation between posttraumatic stress disorder severity and alcohol-related problems. Alcohol Clin. Exp. Res..

[151] Strunk K.K., Lane F.C. (2016). The Beck Depression Inventory, Second Edition (BDI-II): A cross-sample structural analysis. Meas. Eval. Couns. Dev..

[152] Kline A.C., Cooper A.A., Rytwinski N.K., Feeny N.C. (2021). The effect of concurrent depression on PTSD outcomes in trauma-focused psychotherapy: A meta-analysis of randomized controlled trials. Behav. Ther..

[153] Brouwer D., Meijer R.R., Zevalkink J. (2013). On the factor structure of the Beck Depression Inventory-II: G Is the Key. Psychol. Assess..

[154] Faro A., Pereira C.R. (2020). Factor structure and gender invariance of the Beck Depression Inventory–Second Edition (BDI-II) in a community-dwelling sample of adults. Health Psychol. Behav. Med..

[155] Keller F., Kirschbaum-Lesch I., Straub J. (2020). Factor structure and measurement invariance across gender of the Beck Depression Inventory-II in adolescent psychiatric patients. Front. Psychiatry.

[156] McElroy E., Casey P., Adamson G., Filippopoulos P., Shevlin M. (2018). A comprehensive analysis of the factor structure of the Beck Depression Inventory-II in a sample of outpatients with adjustment disorder and depressive episode. Ir. J. Psychol. Med..

[157] Young K.S., Bookheimer S.Y., Nusslock R., Zinbarg R.E., Damme K.S.F., Chat I.K.-Y., Kelley N.J., Vinograd M., Perez M., Chen K. (2021). Dysregulation of threat neurocircuitry during fear extinction: The role of anhedonia. Neuropsychopharmacology.

[158] Jakši N., Brajkovic L., Ivezic E., Topic R., Jakovljevic M. (2012). The role of personality traits in posttraumatic stress disorder (PTSD). Psychiatr. Danub..

[159] Rooney E.A., Hallauer C.J., Xie H., Shih C.-H., Rapport D., Elhai J.D., Wang X. (2022). Longitudinal PTSD symptom trajectories: Relative contributions of state anxiety, depression, and emotion dysregulation. J. Affect. Disord..

[160] Asmundson G.J.G., Stapleton J.A. (2008). Associations between dimensions of anxiety sensitivity and PTSD symptom clusters in active-duty police officers. Cogn. Behav. Ther..

[161] Marshall G.N., Miles J.N.V., Stewart S.H. (2010). Anxiety sensitivity and PTSD symptom severity are reciprocally related: Evidence from a longitudinal study of physical trauma survivors. J. Abnorm. Psychol..

[162] Simpson T., Jakupcak M., Luterek J.A. (2006). Fear and avoidance of internal experiences among patients with substance use disorders and PTSD: The centrality of anxiety sensitivity. J. Trauma. Stress.

[163] Gutner C.A., Nillni Y.I., Suvak M., Wiltsey-Stirman S., Resick P.A. (2013). Longitudinal course of anxiety sensitivity and PTSD symptoms in cognitive-behavioral therapies for PTSD. J. Anxiety Disord..

[164] Manzar M.D., BaHammam A.S., Hameed U.A., Spence D.W., Pandi-Perumal S.R., Moscovitch A., Streiner D.L. (2018). Dimensionality of the pittsburgh sleep quality index: A systematic review. Health Qual. Life Outcomes.

