# Supplementary material for: Predicting Fear Extinction in Posttraumatic Stress Disorder
Source: Brain Sci. 2023 Jul 28;13(8):1131. doi: 10.3390/brainsci13081131 (PMC10452660; doi:10.3390/brainsci13081131)
Supplement: Supplementary file 1 [file brainsci-13-01131-s001.zip › brainsci-2477220-supplementary.pdf]

Supplementary Background

Review of Predictors of Fear Extinction

Trait dissociation [1,2], a dissociative clinical subtype of PTSD [2], trait resilience [3,4], anhedonia symptoms [5–7], and trauma type [8,9] have been found to impact *fear acquisition* and to be relevant to PTSD clinical presentation and treatment outcome [3,4,8,10,11]. Similarly, anxiety sensitivity may impact Pavlovian *fear generalization* [12] and has been associated with PTSD [13–18]. Additionally, trait fearfulness [19], age [20], and race [21] have all been found to be associated with physiological *fear responding* and with PTSD.

Mirroring research on demographic and psychological characteristics, several clinically-relevant measures of baseline physiological activity may predict fear extinction. For example, heart rate variability (HRV) is an index of parasympathetic nervous system activity that may be lower in individuals with PTSD versus controls [22]. Resting HRV has been found to correlate with better fear extinction in healthy humans [23,24]. However, this finding was not replicated by a study using a PTSD sample [25]. It is unclear whether this contrasting finding is best explained by a lack of relationship between HRV and fear extinction in individuals with trauma exposure and/or sample variance [25,26]. Heart rate is a measure of sympathetic nervous system activity, and elevated heart rate has been found to prospectively predict elevated PTSD severity [27,28]. However, we are not aware of studies examining whether baseline heart rate is associated with fear extinction in humans. Baseline startle is a measure of reflexive defensive responding [29] that has been found to predict exposure therapy outcomes in PTSD patients [30]. Further, baseline startle has been found to predict deficient fear extinction in rats [31]. However,

we are not aware of any studies investigating baseline startle as a potential predictor of fear extinction in PTSD.

Supplementary Methods

Selecting Clinical and Self-Report Measures

Overview

We selected scales and subscales to include in our primary and post hoc analyses based on recommendations from the broader statistical literature and theoretical considerations relevant to our study. Following recommendations from the statistical literature [32–35], we sought to reach a compromise between predictive performance, parsimony, and comprehensiveness. Regarding predictive performance of penalized regression models, one important consideration is that risk of overfitting increases as the ratio of sample size to number of candidate predictors decreases (i.e., an increase in the number of predictors can lead to an increase in the risk of overfitting the model) [32–35]. Thus, inclusion of an excessive number of extraneous predictors may lead to overfitting and result in poor predictive performance in samples that were not used to train the algorithm (e.g., the holdout sample in the case of the current study) [32–35]. However, it should also be noted that one strength of penalized regressions is that they are well-suited to predictive modeling problems involving small sample sizes and a large number of predictors (i.e., “large p, small n problems”) [32,34,36]. Another important statistical consideration, related to our univariate regressions, is that an increase in the number of predictors leads to a stricter Bonferroni correction [37]. Thus, inclusion of extraneous predictors increases the risk of type-II errors when examining a large set of Bonferroni-adjusted univariate models [37]. However, a central aim of our study was to examine an array of potential predictors of

extinction in PTSD. To strike a balance between our aim to include a wide range of predictors and the need to protect against overfitting, we selected subscales based on theoretical considerations in relation to our study aims, as described below.

PTSD Symptom Measures

Because the Clinician-Administered PTSD Scale for DSM-5 (CAPS) is the gold-standard measure of PTSD symptom severity [38], we included CAPS measures of total PTSD symptom severity score and of each PTSD symptom cluster. Based on the use of fear extinction as a laboratory model for PTSD symptom remission during treatment [39], we included total CAPS severity in our study. We also included the four DSM-5 CAPS cluster scores based on evidence from multiple studies that they are relevant to fear extinction physiology [40–43]. In addition, FPS in particular has been associated with more severe DSM-IV intrusion symptoms [40,41] and DSM-IV hyperarousal symptoms [42,43]. In addition, our decision to include all four CAPS clusters was informed by the lack of clear consensus regarding which PTSD symptom clusters best predict fear extinction, the lack of prior studies examining the relationship of extinction with DSM-5 PTSD symptom clusters, and the clear clinical relevance of PTSD symptoms to our study aims.

In addition to CAPS total and CAPS symptom clusters, we included the total score from the PTSD Checklist for DSM-5 (PCL-5; [44]) in our primary analyses. Although PCL-5 total score is highly correlated with CAPS total score, these measures are not perfectly concordant [45]. Thus, in light of the potential for differences in operationalization of key variables to alter findings in fear learning research [46–49], we determined that examination of the two most commonly used measures of PTSD symptoms was germane to the literature. We also included

PCL-5 based symptom clusters in post hoc analyses motivated by the goal described above of balancing comprehensiveness with parsimony and statistical considerations.

Trauma Exposure Measures

Given the consistent association of childhood trauma with PTSD and fear learning, we included the Childhood Trauma Questionnaire (CTQ; [50]). Based on literature indicating that childhood trauma is a particularly impactful risk factor for PTSD (for reviews, see [51–54]), we included the CTQ total score in our study. Further, childhood trauma has been found to prospectively predict PTSD treatment response [55]. Additionally, excessive fear physiology and deficient fear extinction may be critical mechanisms linking childhood trauma and PTSD (for review, see [56]. Moreover, translational literature suggests that differences in the *amount* of childhood trauma as well as in qualitatively different *types* of childhood trauma may differentially impact fear physiology [8,9]. The CTQ is well-suited to capturing these effects because it is designed specifically to index both the total exposure as well as the type of exposure (e.g., neglect versus abuse; emotional versus physical versus sexual trauma) [50]. The 5 CTQ subscales (emotional abuse, emotional neglect, physical abuse, physical neglect, and sexual abuse) have been confirmed across multiple factor analytic studies [50,57–60]. Thus, we included total CTQ score and each of the 5 CTQ subscales in our primary analyses.

We also included the experienced+witnessed score and the experienced score from the Life Events Checklist (LEC). The LEC is embedded in the CAPS to screen for exposure to specific potentially traumatic events, using a nominal scale (1 = happened to me, 2 = witnessed it, 3 = learned about it, 4 = part of my job, 5 = not sure; 6 = does not apply). The LEC is used clinically to assess whether potentially traumatic events meet Criterion A for PTSD, prior to conducting the CAPS [61]. Although the LEC was not initially designed as a research tool, it is

widely used in research and several approaches have been used to calculate a measure of trauma exposure using LEC in research [62–64]. Following precedent from our lab [63,65], we included the LEC experienced+witnessed score (i.e., the sum of all events marked with a 1 or a 2) as indicators of Criterion A trauma exposures associated with PTSD. More broadly, the inclusion of this metric is supported by evidence that the probability of developing PTSD from direct exposure (i.e., experienced or witnessed events) is higher than the probability of developing the disorder from indirect exposure [62]. Based on prior research that LEC directly experienced events (i.e., the sum of all events marked with a 1) are the most reliably reported and the strongest predictors of PTSD [64,66], we also included the LEC experienced score in our analyses.

Dissociation Measures

We included measures of dissociation due to its central importance for understanding clinical heterogeneity and treatment response within PTSD (for reviews, see [67–70]) and evidence that it relates to fear learning physiology [1,2,71]. The Dissociative Experiences Scale [72] is the most commonly used measure for studying dissociation in both non-clinical populations and psychiatric disorders, including PTSD [73]. A meta-analysis of clinical studies using the Dissociative Experiences Scale found that, in a comparison of all DSM disorders, PTSD is the disorder with the second highest mean score on the Dissociative Experiences Scale; only dissociative disorder had a higher mean score [73]. Further, the Dissociative Experiences Scale is well suited to our aims because it captures a dimensional continuum of dissociation, which ranges from normative to pathological dissociative experiences, and has high validity and reliability in clinical populations including PTSD [72,73]. In addition to the total score, several factor analytic studies have recommended a 3-factor structure comprising subscales for amnesia,

absorption, and depersonalization/derealization subscales [74–76]. The depersonalization/derealization subscale is particularly relevant to PTSD research because this subscale aligns with the aspects of derealization that are accounted for by the DSM-5 dissociative subtype of PTSD [73]. Thus, there is sufficient evidence and clinical relevance to support inclusion of Dissociative Experiences Scale total score and subscales in our primary analyses.

We included Multiscale Dissociation Inventory (MDI; [77]) and its subscales in our study due to its high relevance to clinically-impairing symptoms in PTSD, but only included it in post hoc analyses due to a high number of missing participants in the training sample. The MDI was completed by the most recent 101 participants and was missing for an additional 4 participants in our training sample. Thus, MDI was missing for 28% of individuals in our training sample (i.e., was missing for 28 out of the first 100 participants in our study) and could not be included in our initial cross-validated predictive models due to excessive missingness [33]. However, to understand the relationship between dissociation and fear extinction, the Multiscale Dissociation Inventory (MDI; [77]) may be necessary to examine in conjunction with the Dissociative Experiences Scale. The MDI is designed to capture clinically-impairing dissociative symptoms (e.g., “Suddenly realizing that hours had gone by and not knowing what you had been doing during that time”) [77]. Prior studies in PTSD have found that dissociation, as measured by the MDI, are related to individual differences in physiology [71] and clinical presentation [78]. Further, MDI total score and several of its subscales have been found to be associated with FPS, SCR, HR, and HRV during fear acquisition [2]. Thus, following up findings that early extinction was predicted by DES and its subscales in our main analyses, we performed Supplementary univariate regression analyses to examine the MDI and its subscales as predictors of early

extinction. All MDI analyses were performed using the subsample of participants for whom MDI was available (n = 97).

Depression Measures

We included the total score on the Beck Depression Inventory–II (BDI; [79]) due to evidence that depression may impact fear extinction in humans [80,81] and evidence that greater depression severity is associated with more severe PTSD as well as worse PTSD treatment outcomes (for meta-analysis, see [82]). Although there is some evidence that multidimensionality may exist in the BDI, the measure was designed as a unidimensional measure and several factor analytic studies have found evidence that a single factor model best fits the data; analyses conducted with subscale scores are not recommended [83–86]. Thus, we did not include BDI subscale scores.

We included the total score from the Snaith Hamilton Pleasure Scale (SHAPS; [87]) due to anhedonia's relevance to PTSD [88] and due to evidence suggesting a link between anhedonia and fear extinction. Recent studies examining an anhedonia-apprehension scale in transdiagnostic anxious-depressed youth have used functional magnetic resonance imaging to show that brain activity during fear extinction learning and recall predicts anhedonia symptoms [89,90]. Although there is some evidence for a 2-factor structure for SHAPS, no clear consensus exists regarding a multi-factor approach [91]. Thus, consistent with the fact that SHAPS was designed as a single measure without subscales [87], we did not include any SHAPS subscales.

Fear and Anxiety Measures

We examined the trait and state subscales of the State-Trait Anxiety Inventory (STAI; [92]) due to their relevance to fear learning [20] and PTSD [93,94]. The STAI was designed to

provide separate measures of trait anxiety (trait subscale) and state anxiety (state subscale), but was not designed to provide an overall score combining those dimensions [92]. Further, both state and trait anxiety have been found to be related to fear physiology in some, but not all, studies (for review see [20]). Moreover, trait anxiety has been found to be positively associated with PTSD (for review see [93]). Further, greater state anxiety has recently been found to be positively correlated with PTSD symptoms at baseline and to prospectively predict a steeper decline in PTSD symptoms over the course of 12 months [94].

We included the total score on the Anxiety Sensitivity Index-3 (ASI; [95]) in our analyses based on evidence and theory linking anxiety sensitivity to physiological conditioned fear responding [12,20] and PTSD clinical presentation [13,14]. The ASI was developed to provide a unidimensional measure of tendency to experience fear due to symptoms of anxiety ("fear of fear") [95]. Some, but not all, studies have found an association between fear learning and anxiety sensitivity (for review, see [20]). Further, a recent study found evidence to suggest that anxiety sensitivity may moderate the relationship between PTSD symptom severity and physiological conditioned fear responding [17]. Additionally, anxiety sensitivity has been found to be associated with PTSD symptom severity [13–15,18,96–98]. Further, anxiety sensitivity and PTSD severity have been found to reciprocally and prospectively predict one another [97]. Moreover, anxiety sensitivity may play an important role in PTSD treatment outcomes [16,99].

We included the total score on the Fear Survey Schedule-II (FSS; [100]) due to its association with fear and PTSD. The FSS was designed as a unidimensional survey to measure tendency to experience fear in response to various real-world stressors and stimuli [100]. Overall score on the FSS taps trait fearfulness and has been associated with physiological fear responding (elevated defensive startle) and with PTSD [19].

Supplementary Materials

Sleep and Resilience Measures

Based on the clear relevance of sleep to PTSD and fear extinction (as reviewed by [101]), we included the total score on the Pittsburgh Sleep Quality Index (PSQI; [102]). The PSQI is a 9-item questionnaire of sleep quality and patterns that was designed to evaluate overall sleep quality [102]. A recent review of factor analytic studies concluded that "the various PSQI factor structures for standard sleep assessment in research and clinical settings may need further validation" [103]. Thus, we did not include any subfactors of PSQI.

We included the total score from the 10 item version of the Connor-Davidson Resilience Scale (CDRISC; [88]) due to demonstrated relevance of resilience to PTSD treatment as well as preliminary evidence that resilience may be related to fear acquisition [3,4]. The 10 item version of the CDRISC is unidimensional and does not have subscales [88].

Supplementary Discussion

Our finding that severity of hyperarousal symptoms (CAPS PTSD Cluster E score) was a predictor of FPS during early extinction is partially consistent with two prior studies but is inconsistent with two other prior studies. In line with our finding, a previous study by Galatzer-Levy et al., (2017) found that a statistically-identified latent subgroup of trauma-exposed adults who had elevated FPS to the CS+ during early extinction also had elevated DSM-IV hyperarousal symptoms [42]. Recently, Richards et al. (2022) found that higher FPS across both conditioned stimuli (CS+ and CS- combined) during early extinction was associated with elevated DSM-IV hyperarousal symptoms [43]. However, in contrast with our finding, two prior studies found that DSM-IV intrusion symptoms, but not DSM-IV hyperarousal symptoms, were associated with FPS to the CS+ during early extinction [40,41]. One possible explanation for these inconsistent findings is that these prior studies used different measures of PTSD symptoms. Specifically, our study and

both prior studies that found an association of early extinction with PTSD hyperarousal symptoms [42,43] used a clinician-administered interview (CAPS for DSM-IV in the case of the other two studies or CAPS for DSM-5 in the case of our study). In contrast, the prior studies finding an association with intrusion symptoms used a self-report measure (i.e., the PSS; [40,41]). Thus, we used a post hoc analysis to explore the association of PTSD symptom clusters with early extinction using a self-report measure that was available in our dataset, the PCL-5. In this study, we found that the finding of an association of early extinction with PTSD hyperarousal symptoms (and no other symptom clusters) was consistent across the two types of PTSD measures. Thus, to our knowledge, this study is the first to demonstrate robustness of an association of PTSD hyperarousal symptom severity with early extinction across clinician-administered and self-reported PTSD measures. This novel finding is noteworthy in light of a recent study showing that fear learning findings in PTSD samples may depend partially on how constructs of interest are measured [47]. However, given that the PSS was not available in our dataset, we cannot rule out that inconsistent findings between prior studies may have been related to the use of PSS in two prior studies [40,41].

Tables and Figures

Table S1.

Beta weights and p-values derived from univariate regressions for predictors of early extinction that were A) significant at the Bonferroni corrected p-value threshold of $p < 0.00098$, B) significant at the traditional threshold for nominal significance of $p < 0.05$, and C) non-significant

A) Bonferroni Significant Predictors (p < 0.001)		
**Variable**	***B***	***p***
FPS to the CS+ during block 3 of *Acquisition*	0.011	< 0.00001
FPS to the CS- during block 3 of *Acquisition*	0.013	< 0.00001
FPS to the CS+ during block 2 of *Acquisition*	0.008	< 0.00001
FPS to the CS- during block 2 of *Acquisition*	0.010	< 0.00001
Dark-Enhanced Startle	0.014	< 0.00001
FPS to the CS+ during block 1 of *Acquisition*	0.007	0.00001
Baseline Startle	0.004	0.00044
**B) Nominally Significant Predictors (p < 0.05)**		
**Variable**	***B***	***p***
Pittsburgh Sleep Quality Index total score	0.077	0.00158
Dissociative Experiences Scale total score	0.026	0.00237
Absorption and Imaginative subscale score of the Dissociative Experiences Scale	0.018	0.00252
Depersonalization and Derealization subscale of the Dissociative Experiences Scale	0.019	0.00465
Trait Anxiety score of the State-Trait Anxiety Inventory	0.022	0.00886
FPS to the CS- during block 1 of *Acquisition*	0.004	0.00959
Cluster E score from the Clinician-Administered PTSD Scale for DSM-5	0.060	0.01200
Female Sex	0.455	0.03130
Beck Depression Inventory total score	0.018	0.04270
**C) Non-Significant Predictors (p > 0.05)**		

**Variable**	***B***	***p***
Emotional Abuse subscale score from the Childhood Trauma Questionnaire	0.030	0.05830
Emotional Neglect subscale score from the Childhood Trauma Questionnaire	0.030	0.08720
Heart Rate during Acquisition	0.013	0.09960
SCR Difference score (CS+ - CS-) during block 1 of Acquisition	0.592	0.10200
Amnesia subscale score of the Dissociative Experiences Scale	0.020	0.12500
Heart Rate during Light blocks of Dark-Enhanced Startle	0.011	0.14300
SCR Difference score (CS+ - CS-) during block 3 of Acquisition	0.795	0.15000
Connor-Davidson Resilience Scale total score	-0.018	0.15700
Heart Rate during Dark blocks of Dark-Enhanced Startle	0.011	0.17300
Clinician-Administered PTSD Scale for DSM-5 total score	0.011	0.19100
Respiratory Sinus Arrhythmia during Baseline	0.100	0.19200
Anxiety Sensitivity Index Total Score	0.009	0.19800
Childhood Trauma Questionnaire total score	0.006	0.21700
Age	-0.013	0.25300
Cluster C score from the Clinician-Administered PTSD Scale for DSM-5	-0.057	0.27100
Heart Rate during Baseline	0.008	0.30000
Snaith–Hamilton Pleasure Scale total score	0.013	0.31300
PTSD Checklist for DSM-5 Total Score	0.006	0.34600
Cluster B score from the Clinician-Administered PTSD Scale for DSM-5	0.023	0.43100
Fear Survey Schedule total score	0.002	0.43900
Ethnicity (White)	-0.153	0.45500
Cluster D score from the Clinician-Administered PTSD Scale for DSM-5	0.013	0.45900
Heart Rate during Dark-Enhanced Startle	-0.030	0.50300
SCR Difference score (CS+ - CS-) during block 2 of Acquisition	0.324	0.54000
Sexual Abuse subscale score from the Childhood Trauma Questionnaire	0.007	0.60600
Physical Abuse subscale score from the Childhood Trauma Questionnaire	0.008	0.65000

Life Events Checklist Experienced+Witnessed	-0.005	0.66800
Respiratory Sinus Arrhythmia during Light blocks of Dark-Enhanced Startle	0.019	0.79600
Physical Neglect subscale score of the Childhood Trauma Questionnaire	-0.005	0.80700
Respiratory Sinus Arrhythmia during Dark blocks of Dark-Enhanced Startle	0.015	0.83700
State Anxiety score of the State-Trait Anxiety Inventory	0.002	0.84100
Respiratory Sinus Arrhythmia during Acquisition	0.013	0.87400
Respiratory Sinus Arrhythmia during Dark-Enhanced Startle	-0.029	0.88100
Life Events Checklist Experienced	0.006	0.88800

Note. *B* = standardized regression coefficient; *p* = p-value

Table S2.

Beta weights, standard errors, t-statistics, and p-values derived from multivariate regressions performed in the whole sample for predictors of early extinction that were identified using: A) cross-validated Lasso and B) cross-validated Elastic Net Regression

**A) Lasso**				
**Variable**	***B***	***SE***	***t***	***p***
Dark-Enhanced Startle	0.011	0.003	4.151	0.00009 ***
Depersonalization and Derealization subscale of the Dissociative Experiences Scale	0.021	0.007	2.938	0.00442 **
FPS to the CS+ during block 3 of Acquisition	0.005	0.002	2.644	0.01001 *
Cluster E score from the Clinician-Administered PTSD Scale for DSM-5	0.064	0.025	2.559	0.01257 *
FPS to the CS+ during block 1 of Acquisition	0.002	0.001	1.814	0.07380
Respiratory Sinus Arrhythmia during Baseline	0.100	0.055	1.799	0.07619
FPS to the CS- during block 2 of Acquisition	0.003	0.002	1.529	0.13069
Anxiety Sensitivity Index Total Score	-0.011	0.007	-1.526	0.13129
Cluster C score from the Clinician-Administered PTSD Scale for DSM-5	-0.067	0.048	-1.416	0.16097
Physical Neglect subscale score of the Childhood Trauma Questionnaire	-0.027	0.02	-1.404	0.16460
FPS to the CS+ during block 2 of Acquisition	0.002	0.002	1.333	0.18678
FPS to the CS- during block 3 of Acquisition	0.002	0.002	0.915	0.36321
Female Sex	0.11	0.171	0.645	0.52068
Pittsburgh Sleep Quality Index total score	0.008	0.026	0.316	0.75262
**B) Elastic Net Regression**				
**Variable**	***B***	***SE***	***t***	***p***
Dark-Enhanced Startle	0.013	0.003	4.637	0.00002 ***
Depersonalization and Derealization subscale of the Dissociative Experiences Scale	0.034	0.012	2.821	0.00654 **
Cluster E score from the Clinician-Administered PTSD Scale for DSM-5	0.07	0.027	2.569	0.01279 *
Physical Neglect subscale score of the Childhood Trauma Questionnaire	-0.062	0.024	-2.55	0.01345 *
FPS to the CS+ during block 1 of Acquisition	0.004	0.002	2.333	0.02316 *
Baseline Startle	0.003	0.001	2.155	0.03536 *
PTSD Checklist for DSM-5 Total Score	-0.014	0.007	-2.029	0.04701 *
FPS to the CS- during block 2 of Acquisition	0.004	0.002	1.853	0.06897
Respiratory Sinus Arrhythmia during Baseline	0.138	0.075	1.85	0.06934
FPS to the CS+ during block 3 of Acquisition	0.003	0.002	1.365	0.17756
Anxiety Sensitivity Index Total Score	-0.011	0.008	-1.361	0.17879
Emotional Neglect subscale score from the Childhood Trauma Questionnaire	0.027	0.022	1.212	0.23032
Dissociative Experiences Scale total score	-0.017	0.015	-1.169	0.24702
Ethnicity (White)	-0.19	0.178	-1.064	0.29188

Emotional Abuse subscale score from the Childhood Trauma Questionnaire	0.019	0.02	0.949	0.34647
FPS to the CS+ during block 2 of Acquisition	0.002	0.002	0.833	0.40805
Female Sex	-0.151	0.199	-0.76	0.4506
Pittsburgh Sleep Quality Index total score	0.018	0.029	0.637	0.5268
Heart Rate during Dark-Enhanced Startle	-0.021	0.041	-0.502	0.61745
FPS to the CS- during block 3 of Acquisition	-0.001	0.003	-0.501	0.61808
Life Events Checklist Experienced + Witnessed	-0.005	0.01	-0.482	0.63178
Heart Rate during Acquisition	0.002	0.008	0.26	0.79588
Cluster C score from the Clinician-Administered PTSD Scale for DSM-5	-0.008	0.051	-0.159	0.87439

Note. Significance codes: 0 ‘***’ 0.001 ‘**’ 0.01 ‘*’ 0.05 ‘.’ 0.1 ‘ ’ 1

Table S3.

Beta weights and p- values derived from univariate regressions for predictors of late extinction that were A) significant at the Bonferroni corrected p-value threshold of p < 0.00098, B) significant at the traditional threshold for nominal significance of p < 0.05, and C) non-significant

A) Bonferroni Significant Predictors (p < 0.001)		
**Variable**	***B***	***p***
Baseline Startle	0.005	< 0.00001
FPS to the CS- during block 3 of *Acquisition*	0.010	< 0.00001
FPS to the CS+ during block 2 of *Acquisition*	0.006	0.00024
FPS to the CS- during block 2 of *Acquisition*	0.006	0.00075
**B) Nominally Significant Predictors (p < 0.05)**		
**Variable**	***B***	***p***
FPS to the CS+ during block 3 of *Acquisition*	0.005	0.00228
FPS to the CS- during block 1 of *Acquisition*	0.004	0.00547
Cluster C score from the Clinician-Administered	-0.131	0.01040
Dark-Enhanced Startle	0.007	0.02330
Physical Neglect subscale score from the Childhood Trauma Questionnaire	-0.049	0.02910
**C) Non-Significant Predictors (p > 0.05)**		
**Variable**	***B***	***p***
Cluster B score from the Clinician-Administered PTSD Scale for DSM-5	-0.054	0.06150
Pittsburgh Sleep Quality Index total score	0.040	0.10700
Absorption and Imaginative subscale score of the Dissociative Experiences Scale	0.010	0.11100
Snaith–Hamilton Pleasure Scale total score	0.020	0.13800
Beck Depression Inventory total score	0.013	0.14000
SCR Difference score (CS+ - CS-) during block 1 of Acquisition	0.523	0.14200
FPS to the CS+ during block 1 of Acquisition	0.002	0.14300
SCR Difference score (CS+ - CS-) during block 2 of Acquisition	0.749	0.14600

Respiratory Sinus Arrhythmia during Dark-Enhanced Startle	-0.282	0.15500
Trait Anxiety score of the State-Trait Anxiety Inventory	0.012	0.16700
Physical Abuse subscale score from the Childhood Trauma Questionnaire	-0.024	0.19400
Dissociative Experiences Scale total score	0.011	0.22100
SCR Difference score (CS+ - CS-) during block 3 of Acquisition	0.639	0.23700
Fear Survey Schedule total score	-0.003	0.24800
Anxiety Sensitivity Index Total Score	0.008	0.25900
Life Events Checklist Experienced + Witnessed	-0.012	0.27000
Childhood Trauma Questionnaire total score	-0.005	0.29100
Cluster E score from the Clinician-Administered PTSD Scale for DSM-5	0.026	0.29300
Ethnicity (White)	0.197	0.33700
Connor-Davidson Resilience Scale total score	-0.012	0.35100
Respiratory Sinus Arrhythmia during Acquisition	0.076	0.37300
Heart Rate during Baseline	-0.007	0.38300
Heart Rate during Acquisition	-0.007	0.40600
Emotional Neglect subscale score from the Childhood Trauma Questionnaire	-0.014	0.43800
Life Events Checklist Experienced	-0.035	0.43900
Respiratory Sinus Arrhythmia during Light blocks of Dark-Enhanced Startle	0.049	0.50800
Heart Rate during Dark-Enhanced Startle	0.029	0.53600
Female Sex	0.135	0.53800
Heart Rate during Light blocks of Dark-Enhanced Startle	-0.005	0.54000
Respiratory Sinus Arrhythmia during Baseline	0.042	0.59800
Heart Rate during Dark blocks of Dark-Enhanced Startle	-0.004	0.61000
Depersonalization and Derealization subscale of the Dissociative Experiences Scale	0.003	0.62400
Age	0.005	0.62500
Clinician-Administered PTSD Scale for DSM-5 total score	-0.004	0.63100

State Anxiety score of the State-Trait Anxiety Inventory	-0.004	0.67000
Sexual Abuse subscale score from the Childhood Trauma Questionnaire	-0.004	0.78400
Cluster D score from the Clinician-Administered PTSD Scale for DSM-5	0.005	0.79600
Amnesia subscale score of the Dissociative Experiences Scale	0.003	0.84300
Respiratory Sinus Arrhythmia during Dark blocks of Dark-Enhanced Startle	0.010	0.89300
Emotional Abuse subscale score from the Childhood Trauma Questionnaire	0.001	0.95000
PTSD Checklist for DSM-5 Total Score	0.000	0.96900

Note. *B* = standardized regression coefficient; *p* = p-value

Table S4.

Beta weights, standard errors, t-statistics, and p-values derived from multivariate regressions performed in the whole sample for predictors of late extinction that were identified using: A) cross-validated Lasso and B) cross-validated Elastic Net Regression

**A) Lasso**				
**Variable**	***B***	***SE***	***t***	***p***
Baseline Startle	0.003	0.001	2.770	0.00708 **
FPS to the CS+ during block 2 of Acquisition	0.004	0.001	2.394	0.01918 *
FPS to the CS- during block 3 of Acquisition	0.005	0.003	1.812	0.07410 .
Physical Neglect subscale score of the Childhood Trauma Questionnaire	-0.048	0.027	-1.750	0.08419 .
Cluster B score from the Clinician-Administered PTSD Scale for DSM-5	-0.053	0.032	-1.645	0.10421
Respiratory Sinus Arrhythmia during Dark-Enhanced Startle	-0.276	0.185	-1.493	0.13964
FPS to the CS- during block 1 of Acquisition	0.002	0.001	1.489	0.14072
SCR Difference score (CS+ - CS-) during block 2 of Acquisition	0.779	0.531	1.469	0.14619
Dark-Enhanced Startle	0.004	0.003	1.353	0.18004
Beck Depression Inventory total score	0.013	0.012	1.067	0.28946
Pittsburgh Sleep Quality Index total score	0.027	0.031	0.884	0.37964
Life Events Checklist Experienced + Witnessed	-0.006	0.011	-0.555	0.58088
Cluster C score from the Clinician-Administered PTSD Scale for DSM-5	-0.030	0.059	-0.521	0.60414
Emotional Neglect subscale score from the Childhood Trauma Questionnaire	0.005	0.022	0.223	0.82447
**B) Elastic Net Regression**				
**Variable**	***B***	***SE***	***t***	***p***
Baseline Startle	0.003	0.001	2.824	0.00666 **
FPS to the CS- during block 3 of Acquisition	0.009	0.003	2.736	0.00845 **
Age	0.033	0.015	2.198	0.03231 *
Cluster C score from the Clinician-Administered PTSD Scale for DSM-5	-0.179	0.084	-2.125	0.03831 *
Cluster E score from the Clinician-Administered PTSD Scale for DSM-5	0.072	0.034	2.107	0.03985 *
FPS to the CS+ during block 3 of Acquisition	-0.004	0.002	-1.674	0.10001
Beck Depression Inventory total score	0.020	0.014	1.406	0.16556
FPS to the CS+ during block 2 of Acquisition	0.003	0.002	1.211	0.23122
Life Events Checklist Experienced + Witnessed	-0.015	0.012	-1.208	0.23230
FPS to the CS- during block 2 of Acquisition	0.004	0.004	1.159	0.25154
Physical Neglect subscale score of the Childhood Trauma Questionnaire	-0.038	0.035	-1.063	0.29253
SCR Difference score (CS+ - CS-) during block 2 of Acquisition	0.722	0.693	1.041	0.30269

FPS to the CS- during block 1 of Acquisition	0.002	0.002	0.946	0.34866
Heart Rate during Acquisition	-0.008	0.009	-0.901	0.37145
Ethnicity (White)	0.183	0.251	0.728	0.46953
Childhood Trauma Questionnaire total score	0.006	0.010	0.626	0.53371
Pittsburgh Sleep Quality Index total score	-0.022	0.038	-0.595	0.55434
Respiratory Sinus Arrhythmia during Dark-Enhanced Startle	-0.115	0.242	-0.474	0.63739
Emotional Neglect subscale score from the Childhood Trauma Questionnaire	-0.010	0.032	-0.333	0.74060
Fear Survey Schedule total score	-0.001	0.003	-0.286	0.77600
Dark-Enhanced Startle	0.001	0.004	0.217	0.82886
Cluster B score from the Clinician-Administered PTSD Scale for DSM-5	-0.004	0.038	-0.098	0.92243

Note. Significance codes: 0 '***' 0.001 '**' 0.01 '*' 0.05 '.' 0.1 ' ' 1

Supplementary Figure S1A

Distribution of FPS in the whole sample during early extinction

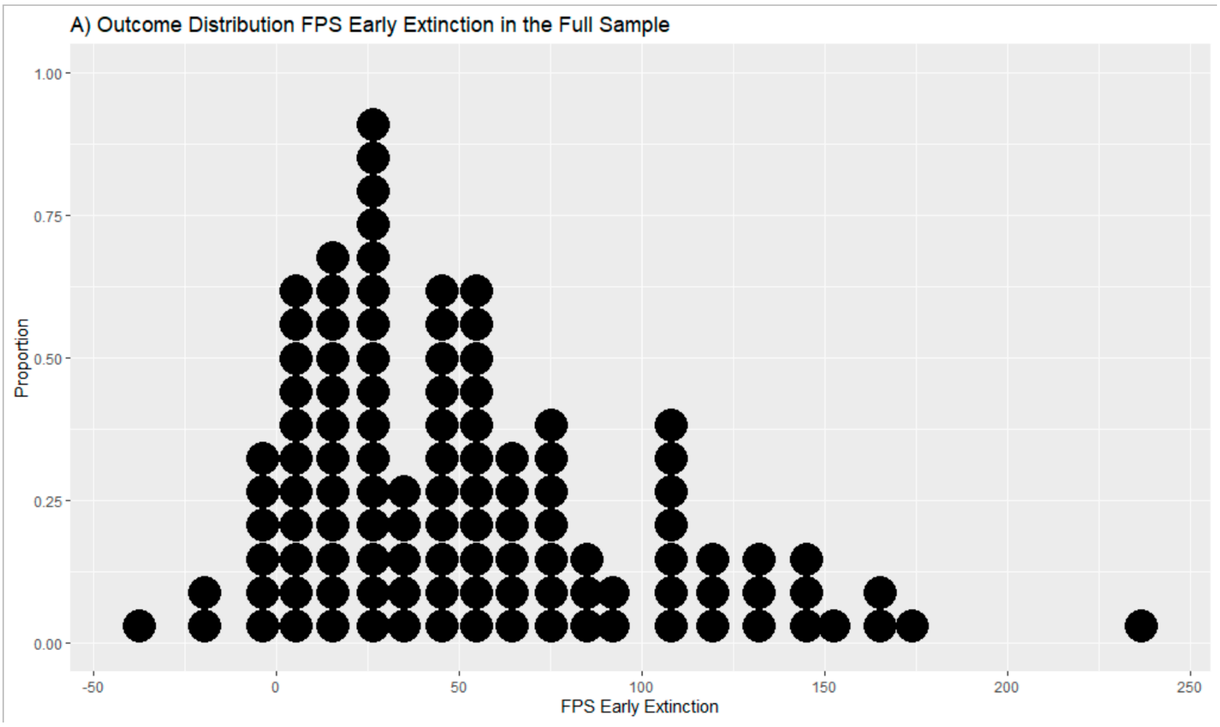

Note. Each dot represents one participant

Supplementary Figure S1B

Distribution of FPS in the training sample during early extinction

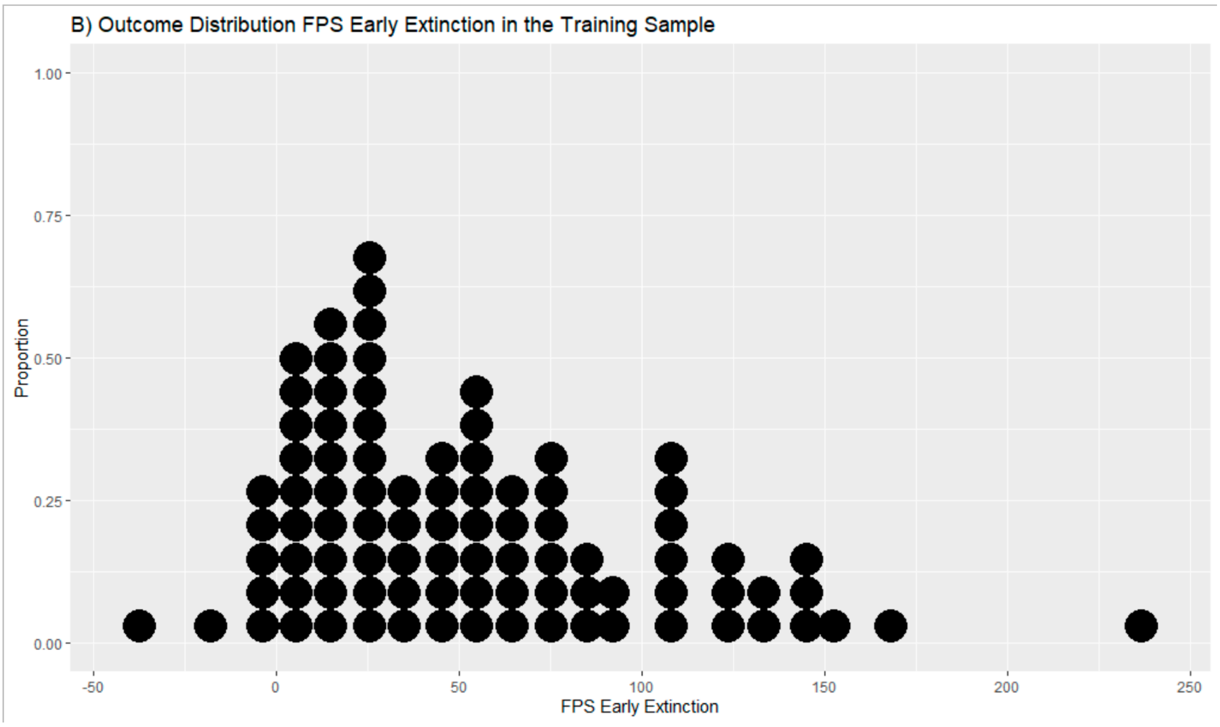

Note. Each dot represents one participant

Supplementary Figure S1C

Distribution of FPS in the holdout sample during early extinction

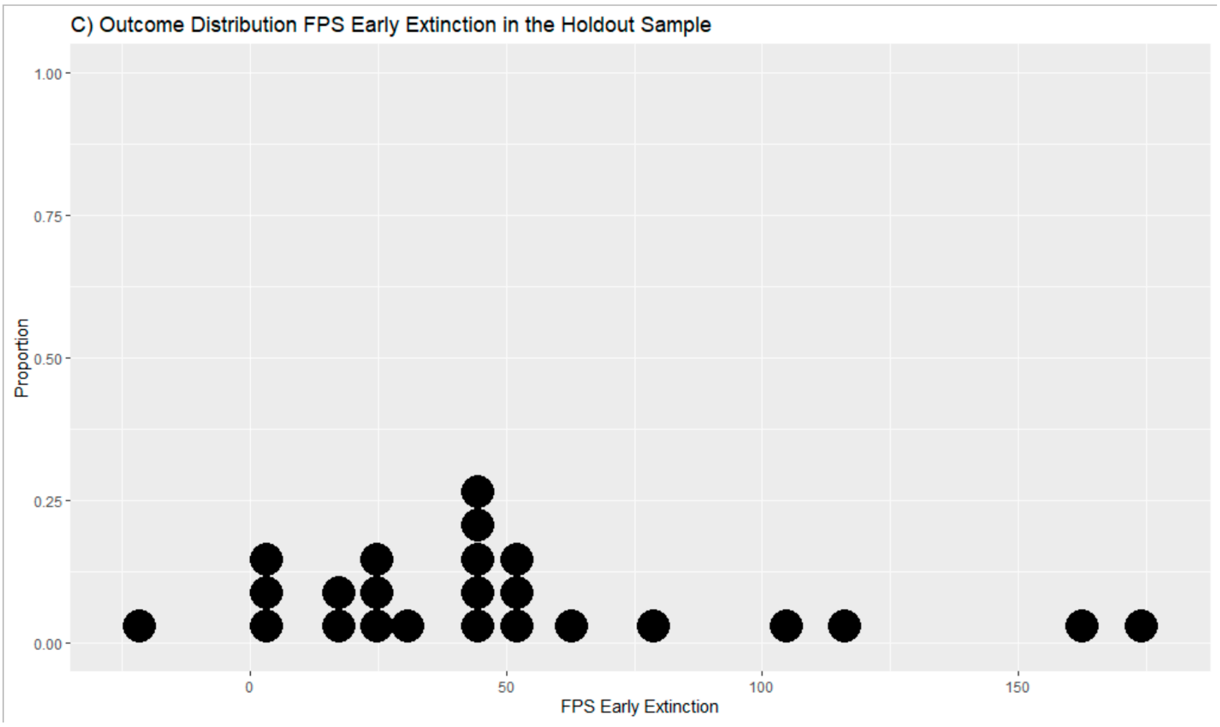

Note. Each dot represents one participant

Supplementary Figure S2A

Distribution of FPS in the whole sample during late extinction.

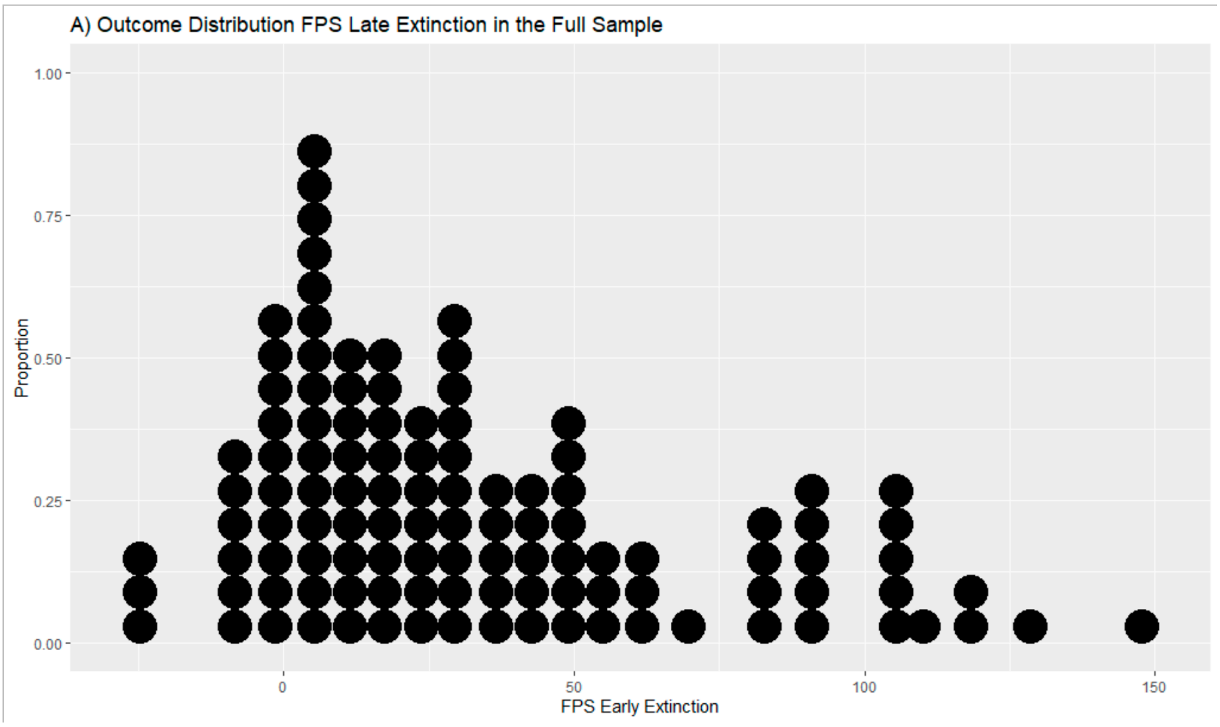

Note. Each dot represents one participant

Supplementary Figure S2B

Distribution of FPS in the training sample during late extinction.

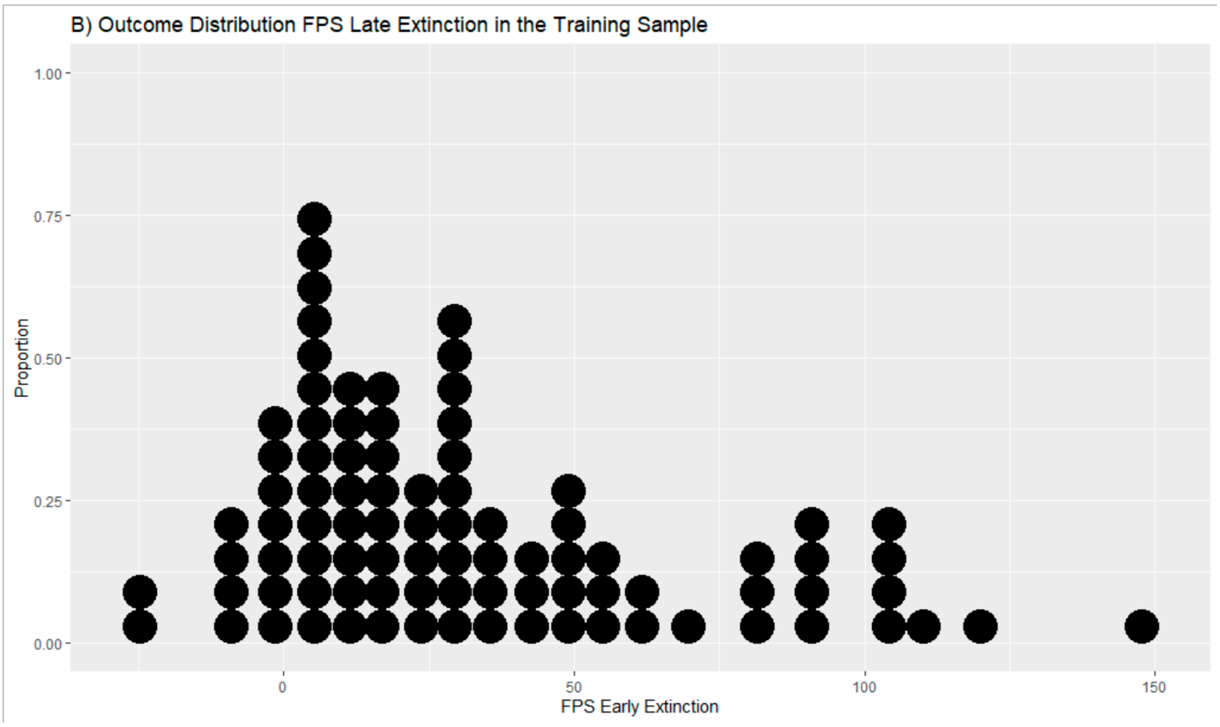

Note. Each dot represents one participant

Supplementary Figure S2C

Distribution of FPS in the holdout sample during late extinction.

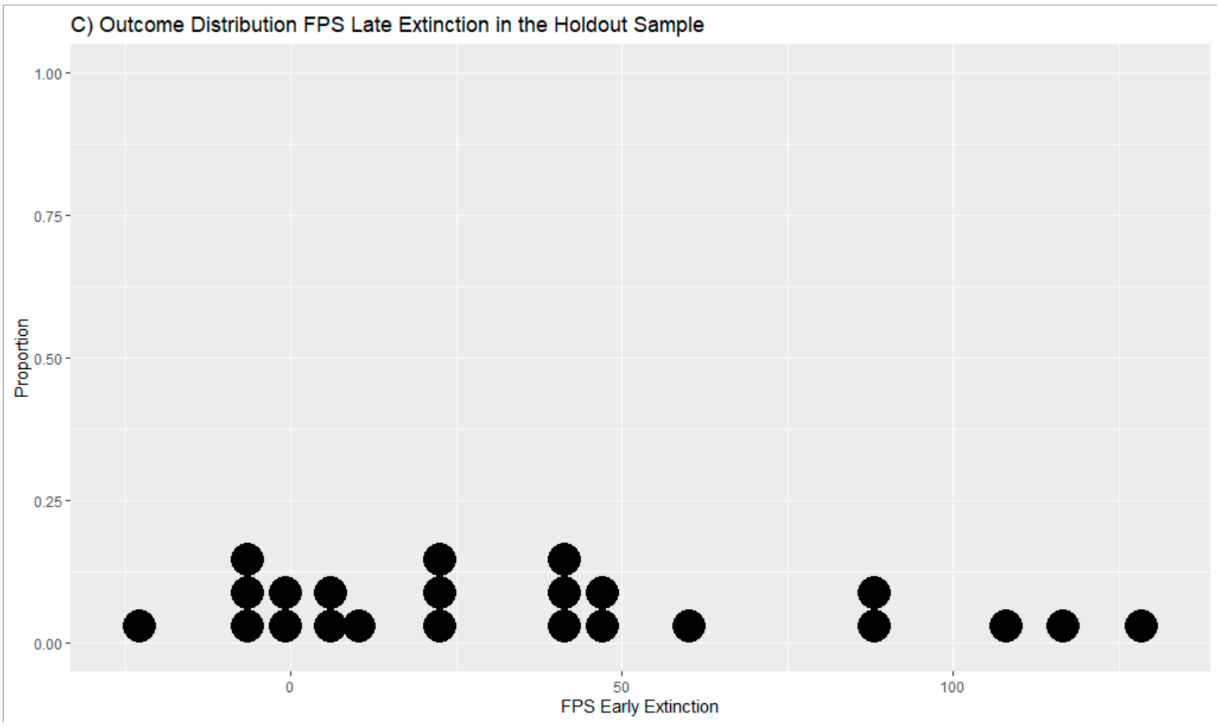

Note. Each dot represents one participant

Supplementary Figure S3.

Heat Map Showing Magnitude of Regression Coefficients and Rank Importance of Predictor Variables for Models Predicting Fear Potentiated Startle During Early Extinction

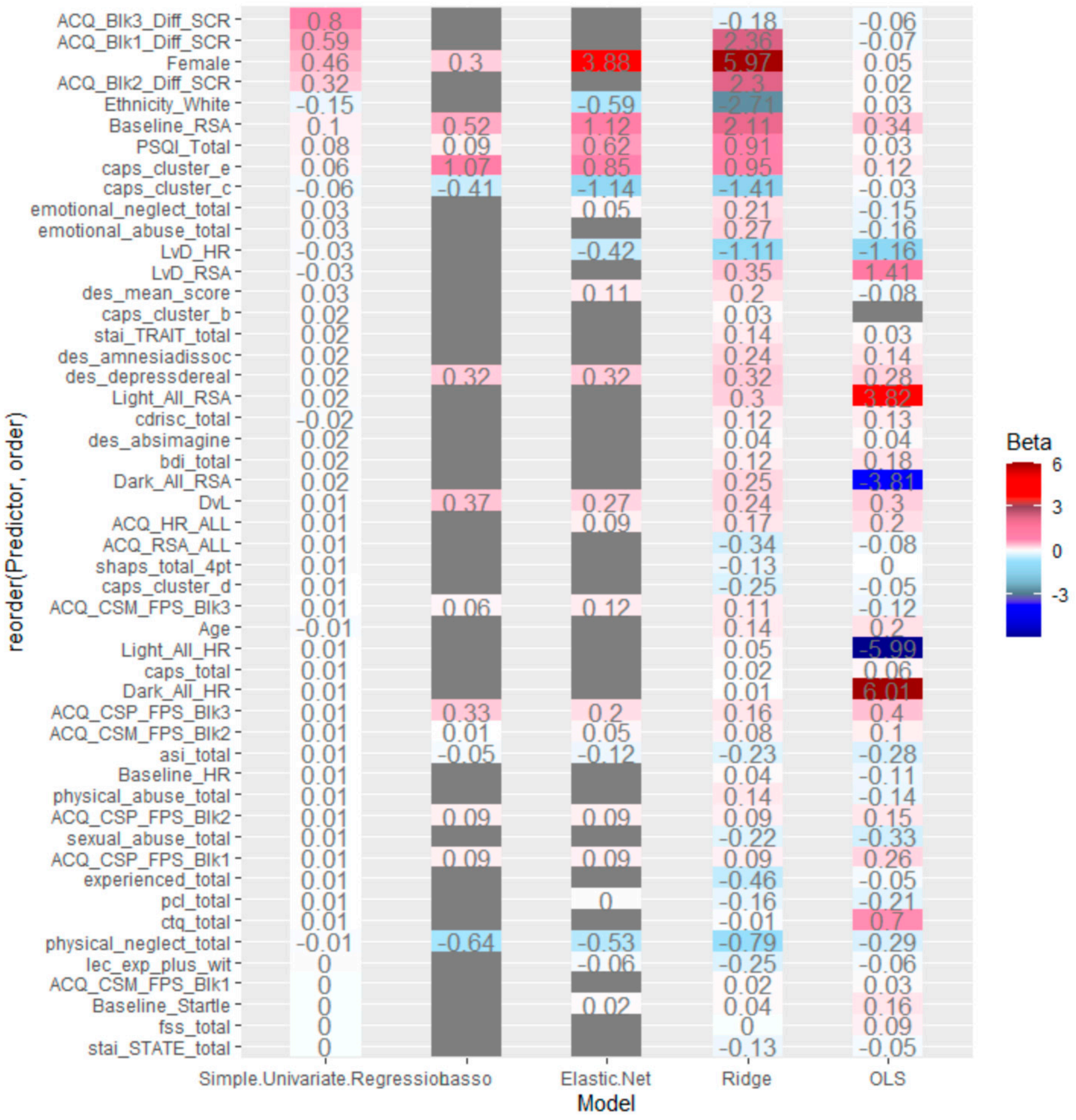

Note. Each cell in column 1 represents one univariate regression model tested in the full sample. For columns 2-5, each column represents the results of one multivariate model trained in the training sample. The numbers in the cells display the beta weight given to each predictor within each model.

Supplementary Figure S4.

Heat Map Showing Size of Regression Coefficients and Rank Importance of Predictor Variables for Models Predicting Fear Potentiated Startle During Late Extinction

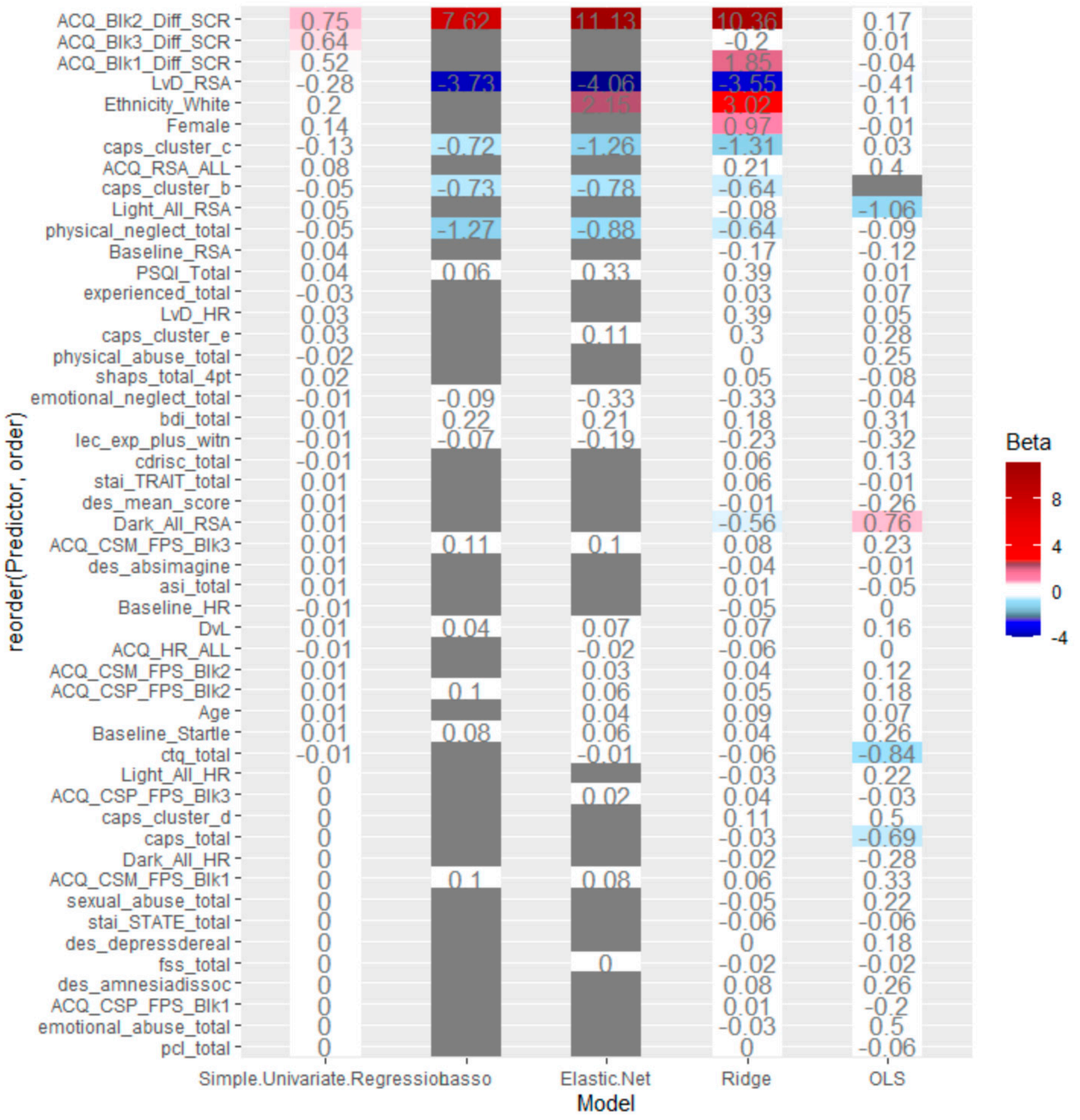

Note. Each cell in column 1 represents one univariate regression model tested in the full sample. For columns 2-5, each column represents the results of one multivariate model trained in the training sample. The numbers in the cells display the beta weight given to each predictor within each model.

Supplementary Materials

References

1. Giesbrecht, T.; Smeets, T.; Merckelbach, H.; Jelicic, M. Depersonalization experiences in undergraduates are related to heightened stress cortisol responses. *J. Nerv. Ment. Dis.* **2007**, *195*, 282–287.

2. Seligowski, A.V.; Lebois, L.A.M.; Hill, S.B.; Kahhale, I.; Wolff, J.D.; Jovanovic, T.; Winternitz, S.R.; Kaufman, M.L.; Ressler, K.J. Autonomic responses to fear conditioning among women with PTSD and dissociation. *Depress. Anxiety* **2019**, *36*, 625–634.

3. van Rooij, S.J.H.; Ravi, M.; Ely, T.D.; Michopoulos, V.; Winters, S.J.; Shin, J.; Marin, M.-F.; Milad, M.R.; Rothbaum, B.O.; Ressler, K.J.; et al. Hippocampal activation during contextual fear inhibition related to resilience in the early aftermath of trauma. *Behav. Brain Res.* **2021**, *408*, 113282.

4. Rakesh, G.; Morey, R.A.; Zannas, A.S.; Malik, Z.; Marx, C.E.; Clausen, A.N.; Kritzer, M.D.; Szabo, S.T. Resilience as a translational endpoint in the treatment of PTSD. *Mol. Psychiatry* **2019**, *24*, 1268–1283.

5. DePierro, J.M.; D'Andrea, W.; Frewen, P. Anhedonia in Trauma Related Disorders: The Good, the Bad, and the Shut-Down. In *Anhedonia: A Comprehensive Handbook Volume II: Neuropsychiatric and Physical Disorders*; Ritsner, M.S., Ed.; Springer Netherlands: Dordrecht, 2014; pp. 175–189 ISBN 978-94-017-8610-2.

6. Olson, E.A.; Kaiser, R.H.; Pizzagalli, D.A.; Rauch, S.L.; Rosso, I.M. Anhedonia in trauma-exposed individuals: functional connectivity and decision-making correlates. *Biol. Psychiatry Cogn. Neurosci. Neuroimaging* **2018**, *3*, 959–967.

7. Walker, R.S.W. The function of conditioned fear in reward propensity: evidence for interrelated approach-avoid systems. Thesis, 2017.

8. Lewis, M.W.; Jones, R.T.; Davis, M.T. Exploring the impact of trauma type and extent of exposure on posttraumatic alterations in 5-HT1A expression. *Transl. Psychiatry* **2020**, *10*, 1–12.

9. Stenson, A.F.; van Rooij, S.J.H.; Carter, S.E.; Powers, A.; Jovanovic, T. A legacy of fear: physiological evidence for intergenerational effects of trauma exposure on fear and safety signal learning among African Americans. *Behav. Brain Res.* **2021**, *402*, 113017.

10. Resick, P.A.; Suvak, M.K.; Johnides, B.D.; Mitchell, K.S.; Iverson, K.M. The Impact of dissociation on PTSD treatment with cognitive processing therapy. *Depress. Anxiety* **2012**, *29*, 718–730.

11. Vinograd, M.; Stout, D.M.; Risbrough, V.B. Anhedonia in Posttraumatic Stress Disorder: Prevalence, Phenotypes, and Neural Circuitry. In *Anhedonia: Preclinical, Translational, and Clinical Integration*; Pizzagalli, D.A., Ed.; Current Topics in Behavioral Neurosciences; Springer International Publishing: Cham, 2022; pp. 185–199 ISBN 978-3-031-09683-9.

12. Hunt, C.; Cooper, S.E.; Hartnell, M.P.; Lissek, S. Anxiety Sensitivity and intolerance of uncertainty facilitate associations between generalized Pavlovian fear and maladaptive avoidance decisions. *J. Abnorm. Psychol.* **2019**, *128*, 315–326.

13. Lebeaut, A.; Tran, J.K.; Vujanovic, A.A. Posttraumatic stress, alcohol use severity, and alcohol use motives among firefighters: the role of anxiety sensitivity. *Addict. Behav.* **2020**, *106*, 106353.

14. Scharff, A.; Ortiz, S.N.; Forrest, L.N.; Smith, A.R. Comparing the clinical presentation of eating disorder patients with and without trauma history and/or comorbid PTSD. *Eat. Disord.* **2021**, *29*, 88–102.

15. Taylor, S.; Koch, W.J.; McNally, R.J. How does anxiety sensitivity vary across the anxiety disorders? *J. Anxiety Disord.* **1992**, *6*, 249–259.

16. Taylor, S. Anxiety sensitivity and its implications for understanding and treating PTSD. *J. Cogn. Psychother.* **2003**, *17*, 179–186.

17. Carpenter, J.K.; Bragdon, L.; Pineles, S.L. Conditioned physiological reactivity and ptsd symptoms across the menstrual cycle: anxiety sensitivity as a moderator. *Psychol. Trauma Theory Res. Pract. Policy* **2022**, *14*, 453–461.

18. Overstreet, C.; Brown, E.; Berenz, E.C.; Brown, R.C.; Hawn, S.; McDonald, S.; Pickett, T.; Danielson, C.K.; Thomas, S.; Amstadter, A. Anxiety sensitivity and distress tolerance typologies and relations to posttraumatic stress disorder: A cluster analytic approach. *Mil. Psychol. Off. J. Div. Mil. Psychol. Am. Psychol. Assoc.* **2018**, *30*, 547–556.

19. Vaidyanathan, U.; Patrick, C.J.; Bernat, E.M. Startle reflex potentiation during aversive picture viewing as an indicator of trait fear. *Psychophysiology* **2009**, *46*, 75–85.

20. Lonsdorf, T.B.; Merz, C.J. More than Just Noise: Inter-Individual differences in fear acquisition, extinction and return of fear in humans - biological, experiential, temperamental factors, and methodological pitfalls. *Neurosci. Biobehav. Rev.* **2017**, *80*, 703–728.

21. Alexandra Kredlow, M.; Orr, S.P.; Otto, M.W. Who is studied in de novo fear conditioning paradigms? an examination of demographic and stimulus characteristics predicting fear learning. *Int. J. Psychophysiol. Off. J. Int. Organ. Psychophysiol.* **2018**, *130*, 21–28.

22. Beauchaine, T.P.; Thayer, J.F. Heart rate variability as a transdiagnostic biomarker of psychopathology. *Int. J. Psychophysiol.* **2015**, *98*, 338–350.

23. Pappens, M.; Schroijen, M.; Sütterlin, S.; Smets, E.; Bergh, O.V. den; Thayer, J.F.; Diest, I.V. Resting Heart rate variability predicts safety learning and fear extinction in an interoceptive fear conditioning paradigm. *PLOS ONE* **2014**, *9*, e105054.

24. Wendt, J.; Neubert, J.; Koenig, J.; Thayer, J.F.; Hamm, A.O. Resting heart rate variability is associated with inhibition of conditioned fear. *Psychophysiology* **2015**, *52*, 1161–1166.

25. Seligowski, A.V.; Lee, D.J.; Miron, L.R.; Orcutt, H.K.; Jovanovic, T.; Norrholm, S.D. Prospective associations between emotion dysregulation and fear-potentiated startle: the moderating effect of respiratory sinus arrhythmia. *Front. Psychol.* **2016**, *7*, 652.

26. Button, K.S.; Ioannidis, J.P.A.; Mokrysz, C.; Nosek, B.A.; Flint, J.; Robinson, E.S.J.; Munafò, M.R. Power failure: why small sample size undermines the reliability of neuroscience. *Nat. Rev. Neurosci.* **2013**, *14*, 365–376.

27. Morris, M.C.; Hellman, N.; Abelson, J.L.; Rao, U. Cortisol, heart rate, and blood pressure as early markers of PTSD Risk: A systematic review and meta-analysis. *Clin. Psychol. Rev.* **2016**, *49*, 79–91.

28. Schiweck, C.; Piette, D.; Berckmans, D.; Claes, S.; Vrieze, E. Heart rate and high frequency heart rate variability during stress as biomarker for clinical depression. a systematic review. *Psychol. Med.* **2019**, *49*, 200–211.

29. Bradford, D.E.; Kaye, J.T.; Curtin, J.J. Not just noise: individual differences in general startle reactivity predict startle response to uncertain and certain threat. *Psychophysiology* **2014**, *51*, 407–411.

30. Norrholm, S.D.; Jovanovic, T.; Gerardi, M.; Breazeale, K.G.; Price, M.; Davis, M.; Duncan, E.; Ressler, K.J.; Bradley, B.; Rizzo, A.; et al. Baseline psychophysiological and cortisol reactivity as a predictor of PTSD treatment outcome in virtual reality exposure therapy. *Behav. Res. Ther.* **2016**, *82*, 28–37.

31. Russo, A.S.; Parsons, R.G. Acoustic Startle Response in Rats Predicts Inter-Individual Variation in Fear Extinction. *Neurobiol. Learn. Mem.* **2017**, *139*, 157–164.

32. Ambler, G.; Seaman, S.; Omar, R.Z. An evaluation of penalised survival methods for developing prognostic models with rare events. *Stat. Med.* **2012**, *31*, 1150–1161, doi:10.1002/sim.4371.

33. Mohri, M.; Rostamizadeh, A.; Talwalkar, A. *Foundations of Machine Learning, Second Edition*; MIT Press, 2018; ISBN 978-0-262-35136-2.

34. Pavlou, M.; Ambler, G.; Seaman, S.R.; Guttmann, O.; Elliott, P.; King, M.; Omar, R.Z. How to develop a more accurate risk prediction model when there are few events. *BMJ* **2015**, *351*, h3868.

35. Van Calster, B.; van Smeden, M.; De Cock, B.; Steyerberg, E.W. Regression shrinkage methods for clinical prediction models do not guarantee improved performance: simulation study. *Stat. Methods Med. Res.* **2020**, *29*, 3166–3178.

36. Li, X.; Liang, C.; Ma, F. Forecasting Stock market volatility with a large number of predictors: new evidence from the MS-MIDAS-LASSO Model. *Ann. Oper. Res.* **2022,** *26*, 1-40.

37. VanderWeele, T.J.; Mathur, M.B. Some desirable properties of the Bonferroni correction: is the Bonferroni correction really so bad? *Am. J. Epidemiol.* **2019**, *188*, 617–618.

38. Weathers, F.W.; Bovin, M.J.; Lee, D.J.; Sloan, D.M.; Schnurr, P.P.; Kaloupek, D.G.; Keane, T.M.; Marx, B.P. The Clinician-Administered PTSD Scale for DSM–5 (CAPS-5): Development and initial psychometric evaluation in military veterans. *Psychol. Assess.* **2018**, *30*, 383–395.

39. Zuj, D.V.; Palmer, M.A.; Lommen, M.J.J.; Felmingham, K.L. The centrality of fear extinction in linking risk factors to PTSD: A narrative review. *Neurosci. Biobehav. Rev.* **2016**, *69*, 15–35.

40. Norrholm, S.D.; Jovanovic, T.; Olin, I.W.; Sands, L.A.; Karapanou, I.; Bradley, B.; Ressler, K.J. Fear extinction in traumatized civilians with posttraumatic stress disorder: relation to symptom severity. *Biol. Psychiatry* **2011**, *69*, 556–563.

41. Norrholm, S.D.; Glover, E.M.; Stevens, J.S.; Fani, N.; Galatzer-Levy, I.R.; Bradley, B.; Ressler, K.J.; Jovanovic, T. Fear Load: The psychophysiological over-expression of fear as an intermediate phenotype associated with trauma reactions. *Int. J. Psychophysiol.* **2015**, *98*, 270–275.

42. Galatzer-Levy, I.R.; Andero, R.; Sawamura, T.; Jovanovic, T.; Papini, S.; Ressler, K.J.; Norrholm, S.D. A Cross species study of heterogeneity in fear extinction learning in relation to FKBP5 variation and expression: implications for the acute treatment of posttraumatic stress disorder. *Neuropharmacology* **2017**, *116*, 188–195, doi:10.1016/j.neuropharm.2016.12.023.

43. Richards, A.; Inslicht, S.S.; Yack, L.M.; Metzler, T.J.; Russell Huie, J.; Straus, L.D.; Dukes, C.; Hubachek, S.Q.; Felmingham, K.L.; Mathalon, D.H.; et al. The Relationship of Fear-potentiated startle and polysomnography-measured sleep in trauma-exposed men and women with and without PTSD: Testing REM sleep effects and exploring the roles of an integrative measure of sleep, PTSD Symptoms, and biological sex. *Sleep* **2022**, *45*, zsab271.

44. Blevins, C.A.; Weathers, F.W.; Witte, T.K. Dissociation and Posttraumatic Stress Disorder: A latent profile analysis. *J. Trauma. Stress* **2014**, *27*, 388–396.

45. Lee, D.J.; Weathers, F.W.; Thompson-Hollands, J.; Sloan, D.M.; Marx, B.P. Concordance in PTSD symptom change between DSM-5 versions of the Clinician-Administered PTSD Scale (CAPS-5) and PTSD Checklist (PCL-5). *Psychol. Assess.* **2022**, *34*, 604–609.

46. Kuhn, M.; Gerlicher, A.M.V.; Lonsdorf, T.B. Navigating the Manyverse of Skin Conductance Response Quantification Approaches – A Direct comparison of trough-to-peak, baseline correction, and model-based approaches in Ledalab and PsPM. *Psychophysiology* **2022**, *59,* e14058.

47. Lewis, M.W.; Bradford, D.E.; Pace-Schott, E.F.; Rauch, S.L.; Rosso, I.M. Multiverse analyses of fear acquisition and extinction retention in posttraumatic stress disorder. *Psychophysiology* ***2023**, 60, e14265.*

48. Lonsdorf, T.B.; Gerlicher, A.; Klingelhöfer-Jens, M.; Krypotos, A.-M. Multiverse analyses in fear conditioning research. *Behav. Res. Ther.* **2022**, *153*, 104072.

49. Sjouwerman, R.; Scharfenort, R.; Lonsdorf, T.B. Individual differences in fear acquisition: multivariate analyses of different emotional negativity scales, physiological responding, subjective measures, and neural activation. *Sci. Rep.* **2020**, *10*, 15283.

50. Bernstein, D.P.; Fink, L.; Handelsman, L.; Foote, J. *Childhood Trauma Questionnaire*; APA PsycTests; APA: Washington, DC, USA, **1994**.

51. Cross, D.; Fani, N.; Powers, A.; Bradley, B. Neurobiological development in the context of childhood trauma. *Clin. Psychol. Sci. Pract.* **2017**, *24*, 111–124.

52. Heim, C.; Nemeroff, C.B. The role of childhood trauma in the neurobiology of mood and anxiety disorders: preclinical and clinical Studies. *Biol. Psychiatry* **2001**, *49*, 1023–1039.

53. Herringa, R.J. Trauma, PTSD, and the Developing Brain. *Curr. Psychiatry Rep.* **2017**, *19*, 69.

54. Nemeroff, C.B. Neurobiological Consequences of Childhood Trauma. *J Clin Psychiatry*. **2004**, *65* Suppl 1:18-28.

55. Bosch, J.; Mackintosh, M.-A.; Wells, S.Y.; Wickramasinghe, I.; Glassman, L.H.; Morland, L.A. PTSD treatment response and quality of life in women with childhood trauma histories. *Psychol. Trauma Theory Res. Pract. Policy* **2020**, *12*, 55–63.

56. McLaughlin, K.A.; Lambert, H.K. Child trauma exposure and psychopathology: mechanisms of risk and resilience. *Curr. Opin. Psychol.* **2017**, *14*, 29–34.

57. Charak, R.; de Jong, J.T.V.M.; Berckmoes, L.H.; Ndayisaba, H.; Reis, R. Assessing the factor structure of the Childhood Trauma Questionnaire, and cumulative effect of abuse and neglect on mental health among adolescents in conflict-affected Burundi. *Child Abuse Negl.* **2017**, *72*, 383–392.

58. Cheng, Y.-C.; Kuo, P.-H. Reliability and factor structure of the Chinese version of Childhood Trauma Questionnaire-Short Form in patients with substance use disorder. *Taiwanese J. Psychiatry (Taipei).* **2018** 32, 52-62.

59. Scher, C.D.; Stein, M.B.; Asmundson, G.J.; McCreary, D.R.; Forde, D.R. The Childhood Trauma Questionnaire in a community sample: psychometric properties and normative data. *J. Trauma. Stress* **2001**, *14*, 843–857.

60. Spinhoven, P.; Penninx, B.W.; Hickendorff, M.; van Hemert, A.M.; Bernstein, D.P.; Elzinga, B.M. Childhood Trauma Questionnaire: Factor structure, measurement invariance, and validity across emotional disorders. *Psychol. Assess.* **2014**, *26*, 717–729.

61. Gray, M.J.; Litz, B.T.; Hsu, J.L.; Lombardo, T.W. Psychometric properties of the Life Events Checklist. *Assessment* **2004**, *11*, 330–341.

62. May, C.L.; Wisco, B.E. Defining Trauma: How level of exposure and proximity affect risk for posttraumatic stress disorder. *Psychol. Trauma Theory Res. Pract. Policy* **2016**, *8*, 233–240.

63. Olson, E.A.; Overbey, T.A.; Ostrand, C.G.; Pizzagalli, D.A.; Rauch, S.L.; Rosso, I.M. Childhood maltreatment experiences are associated with altered diffusion in occipito-temporal white matter Pathways. *Brain Behav.* **2019**, *10*, e01485.

64. Weis, C.N.; Webb, E.K.; Stevens, S.K.; Larson, C.L.; deRoon-Cassini, T.A. Scoring the Life Events Checklist: comparison of three scoring methods. *Psychol. Trauma Theory Res. Pract. Policy* **2022**, *14*, 714–720.

65. Rosso, I.M.; Silveri, M.M.; Olson, E.A.; Eric Jensen, J.; Ren, B. Regional specificity and clinical correlates of cortical GABA alterations in posttraumatic stress disorder. *Neuropsychopharmacology* **2022**, *47*, 1055–1062.

66. Pugach, C.P.; Nomamiukor, F.O.; Gay, N.G.; Wisco, B.E. Temporal stability of self-reported trauma exposure on the Life Events Checklist for DSM-5. *J. Trauma. Stress* **2021**, *34*, 248–256,.

67. Dorahy, M.J.; van der Hart, O. DSM–5's posttraumatic stress disorder with dissociative symptoms: challenges and future directions. *J. Trauma Dissociation* **2015**, *16*, 7–28.

68. Lanius, R.A.; Vermetten, E.; Loewenstein, R.J.; Brand, B.; Schmahl, C.; Bremner, J.D.; Spiegel, D. Emotion modulation in PTSD: clinical and neurobiological evidence for a dissociative subtype. *Am. J. Psychiatry* **2010**, *167*, 640–647.

69. Lanius, R.A.; Brand, B.; Vermetten, E.; Frewen, P.A.; Spiegel, D. The dissociative subtype of posttraumatic stress disorder: rationale, clinical and neurobiological evidence, and implications. *Depress. Anxiety* **2012**, *29*, 701–708.

70. Schiavone, F.L.; Frewen, P.; McKinnon, M.; Lanius, R.A. The dissociative subtype of PTSD: An update of the literature. **2018**, *29, **2018**,* 2-13.

71. Powers, A.; Mekawi, Y.; Fickenwirth, M.; Nugent, N.R.; Dixon, H.D.; Minton, S.; Kim, Y.J.; Gluck, R.; Carter, S.; Fani, N.; et al. Emotion Dysregulation and dissociation contribute to decreased heart rate variability to an acute psychosocial stressor in trauma-exposed black women. *J. Psychiatr. Res.* **2021**, *142*, 125–131.

72. Carlson, E.B.; Putnam, F.W. An Update on the Dissociative Experiences Scale. *Dissociation Prog. Dissociative Disord.* **1993**, *6*, 16–27.

73. Lyssenko, L.; Schmahl, C.; Bockhacker, L.; Vonderlin, R.; Bohus, M.; Kleindienst, N. Dissociation in psychiatric disorders: a meta-analysis of studies using the Dissociative Experiences Scale. *Am. J. Psychiatry* **2018**, *175*, 37–46.

74. Carlson, E.B.; Rosser-Hogan, R. Trauma experiences, Posttraumatic Stress, dissociation, and depression in Cambodian refugees. *Am. J. Psychiatry* **1991**, *148*, 1548–1551.

75. Ross, C.A.; Ellason, J.W.; Anderson, G. A factor analysis of the Dissociative Experiences Scale (DES) in dissociative identity disorder. *Dissociation Prog. Dissociative Disord.* **1995**, *8*, 229–235.

76. Stockdale, G.D.; Gridley, B.E.; Balogh, D.W.; Holtgraves, T. Confirmatory factor analysis of single- and multiple-factor competing models of the dissociative experiences scale in a nonclinical sample. *Assessment* **2002**, *9*, 94–106.

77. Briere, J.; Weathers, F.W.; Runtz, M. Is Dissociation a multidimensional construct? data from the Multiscale Dissociation Inventory. *J. Trauma. Stress* **2005**, *18*, 221–231.

78. Patel, H.; O'Connor, C.; Andrews, K.; Amlung, M.; Lanius, R.; McKinnon, M.C. Dissociative symptomatology mediates the relation between posttraumatic stress disorder severity and alcohol-related problems. *Alcohol Clin. Exp. Res.* **2022**, *46*, 289–299.

79. Strunk, K.K.; Lane, F.C. The Beck Depression Inventory, Second Edition (BDI-II): A cross-sample structural analysis. *Meas. Eval. Couns. Dev.* **2016**, 0748175616664010.

80. Kuhn, M.; Höger, N.; Feige, B.; Blechert, J.; Normann, C.; Nissen, C. Fear extinction as a model for synaptic plasticity in Major Depressive Disorder. *PLoS ONE* **2014**, *9*, e115280.

81. Rainer, C.; Nasrouei, S.; Tschofen, S.; Bliem, H.R.; Wilhelm, F.H.; Marksteiner, J. Fear acquisition and extinction in elderly patients with depression. *J. Affect. Disord.* **2020**, *276*, 197–204.

82. Kline, A.C.; Cooper, A.A.; Rytwinski, N.K.; Feeny, N.C. The effect of concurrent depression on ptsd outcomes in trauma-focused psychotherapy: a meta-analysis of randomized controlled trials. *Behav. Ther.* **2021**, *52*, 250–266.

83. Brouwer, D.; Meijer, R.R.; Zevalkink, J. On the factor structure of the Beck Depression Inventory-II: G Is the Key. *Psychol. Assess.* **2013**, *25*, 136–145.

84. Faro, A.; Pereira, C.R. Factor structure and gender invariance of the Beck Depression Inventory – Second Edition (BDI-II) in a community-dwelling sample of adults. *Health Psychol. Behav. Med.* **2020**, *8*, 16–31.

85. Keller, F.; Kirschbaum-Lesch, I.; Straub, J. Factor structure and measurement invariance across gender of the Beck Depression Inventory-II in adolescent psychiatric patients. *Front. Psychiatry* **2020**, *11*, 527559.

86. McElroy, E.; Casey, P.; Adamson, G.; Filippopoulos, P.; Shevlin, M. A comprehensive analysis of the factor structure of the Beck Depression Inventory-II in a sample of outpatients with adjustment disorder and depressive episode. *Ir. J. Psychol. Med.* **2018**, *35*, 53–61.

87. Snaith, R.P.; Hamilton, M.; Morley, S.; Humayan, A.; Hargreaves, D.; Trigwell, P. A scale for the assessment of hedonic tone the Snaith-Hamilton Pleasure Scale. *Br. J. Psychiatry J. Ment. Sci.* **1995**, *167*, 99–103.

88. Campbell-Sills, L.; Stein, M.B. Psychometric analysis and refinement of the Connor–Davidson Resilience Scale (CD-RISC): Validation of a 10-item measure of resilience. *J. Trauma. Stress* **2007**, *20*, 1019–1028.

89. Rosenberg, B.M.; Taschereau-Dumouchel, V.; Lau, H.; Young, K.S.; Nusslock, R.; Zinbarg, R.E.; Craske, M.G. A multivoxel pattern analysis of anhedonia during fear extinction: implications for safety learning. *Biol. Psychiatry Cogn. Neurosci. Neuroimaging* **2023**, *8*, 417–425.

90. Young, K.S.; Bookheimer, S.Y.; Nusslock, R.; Zinbarg, R.E.; Damme, K.S.F.; Chat, I.K.-Y.; Kelley, N.J.; Vinograd, M.; Perez, M.; Chen, K.; et al. Dysregulation of threat neurocircuitry during fear extinction: the role of anhedonia. *Neuropsychopharmacology* **2021**, *46*, 1650–1657.

91. Langvik, E.; Borgen Austad, S. Psychometric properties of the Snaith–Hamilton Pleasure Scale and a facet-level analysis of the relationship between anhedonia and extraversion in a nonclinical sample. *Psychol. Rep.* **2019**, *122*, 360–375.

92. Spielberger, C.D. *State-Trait Anxiety Inventory for Adults*; APA PsycTests; APA: Washington, DC, USA, 1983**.**

93. Jakši, N.; Brajkovic, L.; Ivezic, E.; Topic, R.; Jakovljevic, M. The role of personality traits in posttraumatic stress disorder (PTSD). *Psychiatr. Danub.* **2012**, *24*, 256–266.

94. Rooney, E.A.; Hallauer, C.J.; Xie, H.; Shih, C.-H.; Rapport, D.; Elhai, J.D.; Wang, X. Longitudinal PTSD symptom trajectories: relative contributions of state anxiety, depression, and emotion dysregulation. *J. Affect. Disord.* **2022**, *308*, 281–288.

95. Taylor, S.; Zvolensky, M.J.; Cox, B.J.; Deacon, B.; Heimberg, R.G.; Ledley, D.R.; Abramowitz, J.S.; Holaway, R.M.; Sandin, B.; Stewart, S.H.; et al. Robust dimensions of anxiety sensitivity: development and initial validation of the Anxiety Sensitivity Index-3. *Psychol. Assess.* **2007**, *19*.

96. Asmundson, G.J.G.; Stapleton, J.A. Associations between dimensions of anxiety sensitivity and PTSD symptom clusters in active-duty police officers. *Cogn. Behav. Ther.* **2008**, *37*, 66–75.

97. Marshall, G.N.; Miles, J.N.V.; Stewart, S.H. Anxiety sensitivity and PTSD symptom severity are reciprocally related: evidence from a longitudinal study of physical trauma survivors. *J. Abnorm. Psychol.* **2010**, *119*, 143–150.

98. Simpson, T.; Jakupcak, M.; Luterek, J.A. Fear and avoidance of internal experiences among patients with substance use disorders and PTSD: The centrality of anxiety sensitivity. *J. Trauma. Stress* **2006**, *19*, 481–491.

99. Gutner, C.A.; Nillni, Y.I.; Suvak, M.; Wiltsey-Stirman, S.; Resick, P.A. Longitudinal course of anxiety sensitivity and PTSD symptoms in cognitive-behavioral therapies for PTSD. *J. Anxiety Disord.* **2013**, *27*, 728–734.

100. Geer, J.H. The development of a scale to measure fear. *Behav. Res. Ther.* **1965**, *3*, 45–53.

101. Pace-Schott, E.F.; Seo, J.; Bottary, R. The influence of sleep on fear extinction in trauma-related disorders. *Neurobiol. Stress* **2023**, *22*, 100500.

102. Buysse, D.J.; Reynolds, C.F.; Monk, T.H.; Berman, S.R.; Kupfer, D.J. The Pittsburgh sleep quality index: a new instrument for psychiatric practice and research. *Psychiatry Res.* **1989**, *28*, 193–213.

103. Manzar, M.D.; BaHammam, A.S.; Hameed, U.A.; Spence, D.W.; Pandi-Perumal, S.R.; Moscovitch, A.; Streiner, D.L. Dimensionality of the pittsburgh sleep quality index: a systematic review. *Health Qual. Life Outcomes* **2018**, *16*, 89.
